# Evaluating Naphthalene-Modified Metallosalen Complexes as Anticancer Agents

**DOI:** 10.1021/acs.jmedchem.4c03180

**Published:** 2025-07-15

**Authors:** Jemily Acosta-Mercado, Angelica Oliveras-Alsina, Ariana I. Marcano-Maiz, Andrea P. Rivera-Torres, Keysha T. Cordero-Gimenez, Sonya Malavez-Cajigas, Marvin J. Bayro, Marcos J. Ramos-Benitez, Dalice M. Piñero Cruz

**Affiliations:** † Department of Chemistry, 19878 University of Puerto Rico, Rio Piedras Campus, 17 Ave Universidad STE 1701, San Juan, Puerto Rico 00925-2537, United States; ‡ Molecular Science Research Center, Juan Ponce de León STE 1390, San Juan, Puerto Rico 00926, United States; § Department of Basic Sciences, 6650 Ponce Health Sciences University, Ponce, Puerto Rico 00716, United States

## Abstract

The development of novel naphthalene-modified metallosalens incorporating Pt­(II) and Pd­(II) presents a promising approach to addressing limitations in cancer therapies. These metallosalens were synthesized and characterized using single crystal X-ray diffraction (sc-XRD), UV–vis spectroscopy, nuclear magnetic resonance (NMR) spectroscopy, and C, H, N elemental analysis. Their strong metal coordination and enhanced electronic interactions, stemming from π-conjugated aromatic structures, improved their DNA-binding affinity and cytotoxic efficacy. Biological assays confirmed significant cytotoxicity in A375 (melanoma) and H292 (nonsmall cell lung cancer) cell lines, while demonstrating minimal toxicity toward HSAEC healthy lung cells. PtL1 and PtL2 exhibited superior activity, inducing apoptosis with high selectivity for cancer cells, as validated by Incucyte Caspase-3/7 Green Dye assays. These findings highlight the potential of naphthalene-modified metallosalens as broad-spectrum cancer therapies, balancing efficacy with reduced off-target effects, and underscore the importance of ligand design and metal coordination in advancing next-generation chemotherapeutics.

## Graphical abstract

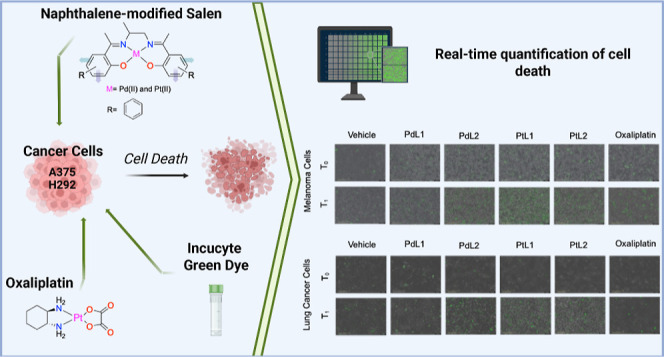

## Introduction

Since the discovery of cisplatin’s anticancer properties, inorganic compounds have received significant attention in biological chemistry.^1^ Widely used platinum-based drugs, including cisplatin, carboplatin, and oxaliplatin,^2^ primarily target DNA.^3^ These drugs inhibit replication or transcription by forming cross-links, ultimately leading to cell death.^4^ However, their lack of selectivity causes severe side effects as healthy tissues are also affected, which limits their clinical utility.^2,5^ These challenges highlight the urgent need for novel anticancer therapies with enhanced efficacy and reduced toxicity, especially as cancer incidence and resistance to current treatments rise.^6,7^

Metal complexes offer advantages over organic compounds, particularly their capacity for fine-tuning geometric and electronic properties.^8,9^ The spatial arrangement of ligands around the metal center and the localized cationic charge significantly influence interactions with biological targets such as DNA or proteins.^8,9^ This adaptability enables the design of metal complexes with potent cytotoxic activity while minimizing off-target effects, positioning them as transformative agents in modern cancer therapies.

Salen-like ligands [*N*,*N*-bis­(salicylidene)-1,2-ethylenediamine], characterized by their strong metal coordination capabilities, have emerged as promising candidates for biologically active compounds (Supporting Information Figure S1). These ligands, containing donor atoms such as nitrogen (N) and oxygen (O), exhibit a wide range of biological functions.^10^ Their straightforward synthesis and adaptable chemical and electronic structures enable the formation of diverse metal complexes, which are particularly valuable in developing anticancer agents. Salen-like metal complexes, or metallosalens, exhibit significant affinity for double-stranded DNA, often causing cleavage and damage—a fundamental mechanism of anticancer activity.^9,11–13^ These planar, π-delocalized systems facilitate stacking interactions with DNA, stabilized by the metal ion, thereby enhancing structural integrity.^9^

Pt­(II) and Pd­(II) metals are particularly relevant in cancer therapy due to their roles in inducing apoptosis through DNA targeting.^14,15^ While Pt­(II) complexes, such as cisplatin, form intrastrand cross-links at guanine-rich sites,^16,18^ Pd­(II) complexes offer similar coordination chemistry with lower toxicity and faster ligand-exchange kinetics,^17^ making them attractive for new drug development.^19,20^ The similarity in their coordination geometries (both d^8^ systems with square-planar arrangements) further underscores their relevance in medicinal chemistry.^21,22^

In this study, we employ a rational design strategy to enhance the DNA-binding properties and anticancer activity of salen-based complexes. By incorporating naphthalene into the framework, we extend the π-conjugated system, facilitating stacking interactions with DNA bases.^9,23,24^ The spatial orientation^9^ of this extended aromatic system is carefully modulated to maintain compound planarity, as deviations due to torsion could impact stacking efficiency and overall binding affinity. Additionally, integrating Pt­(II) and Pd­(II) ions within the salen framework enhances DNA-binding through coordination bonds, disrupting DNA replication via adduct formation.^20^ These combined approaches leverage both structural and electronic factors to optimize interaction with DNA.

This study investigates the synthesis of novel naphthalene-incorporating metallosalens, leveraging their strong metal coordination and π-conjugated systems to improve anticancer efficacy (Figure 1). Pd­(II) and Pt­(II) complexes were synthesized and characterized, and their biological activity was evaluated against A375 (melanoma) and H292 (nonsmall cell lung cancer) cell lines. To assess selectivity, these complexes were also tested on HSAEC healthy lung epithelial cells, providing a comparison of their effects on cancerous and healthy cells. Additionally, their performance was benchmarked against oxaliplatin, a clinically approved platinum-based drug. These findings provide insights into the potential of these metallosalens as selective and effective anticancer agents, highlighting the importance of ligand design and metal coordination in advancing next-generation chemotherapeutics.

**Figure 1:**
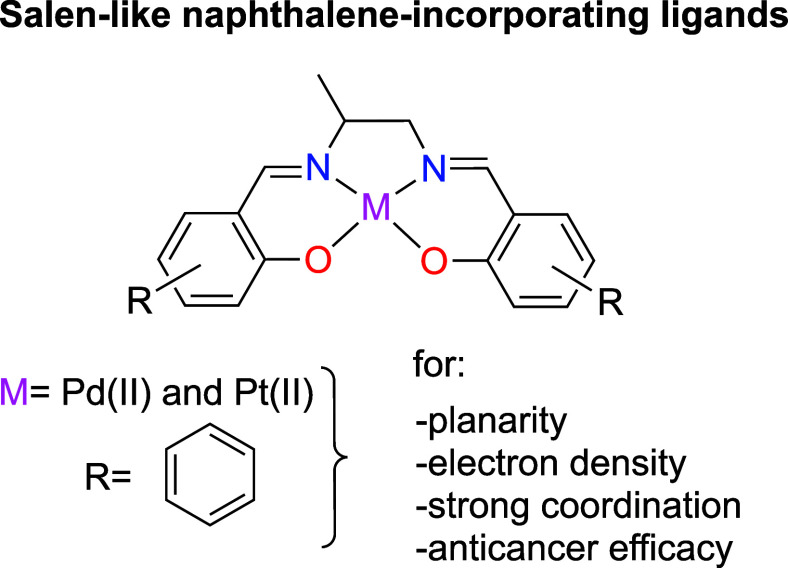
Representation of synthesized naphthalene-incorporating metallosalens. Coordination atoms are highlighted in blue and red, with metals shown in pink.

## Results and Discuission

### Chemistry

The naphthalene-substituted salen ligands, **L1** and **L2**, were prepared using a condensation reaction between a diamine and a hydroxy-substituted aryl ketone, achieving high purity yields of 87% and 83%, respectively (Scheme 1).^25,26^ **L1** corresponds to a previously reported ligand,^25,26^ while **L2** represents a newly synthesized salen-like ligand with similar structural features. Pd­(II) and Pt­(II) complexes were prepared by coordinating these ligands with PdCl_2_ and K_2_PtCl_4_ under inert conditions (see Scheme 1), yielding 63% for **PdL1**, 95% for **PdL2**, 95% for **PtL1**, and 93% for **PtL2**. Salen ligands are characterized by two Schiff bases, which provide a rigid, planar framework linking aromatic moieties and enhancing stability and electronic properties.^25^ Schiff base ligands are of interest due to their straightforward synthesis and their ability to form stable, potent complexes with transition metals.^27^ The inclusion of a naphthalene moiety within the salen ligands not only increases hydrophobicity^28^ but also extends the π-conjugated system, offering two key advantages: (1) enhancing π-stacking interactions with DNA bases, a feature critical for potential intercalation or groove binding, and (2) introducing a specific spatial orientation of the aromatic group that affects the planarity of the compound. This orientation or directionality can induce torsion in the structure, which may influence stacking interactions with DNA. These structural modifications, combined with the integration of Pt­(II) and Pd­(II) ions within the salen framework, are expected to improve the cytotoxic efficacy of these metallosalens, similar to platinum-based drugs that interact with DNA to induce apoptosis in cancer cells.

**Scheme 1:**
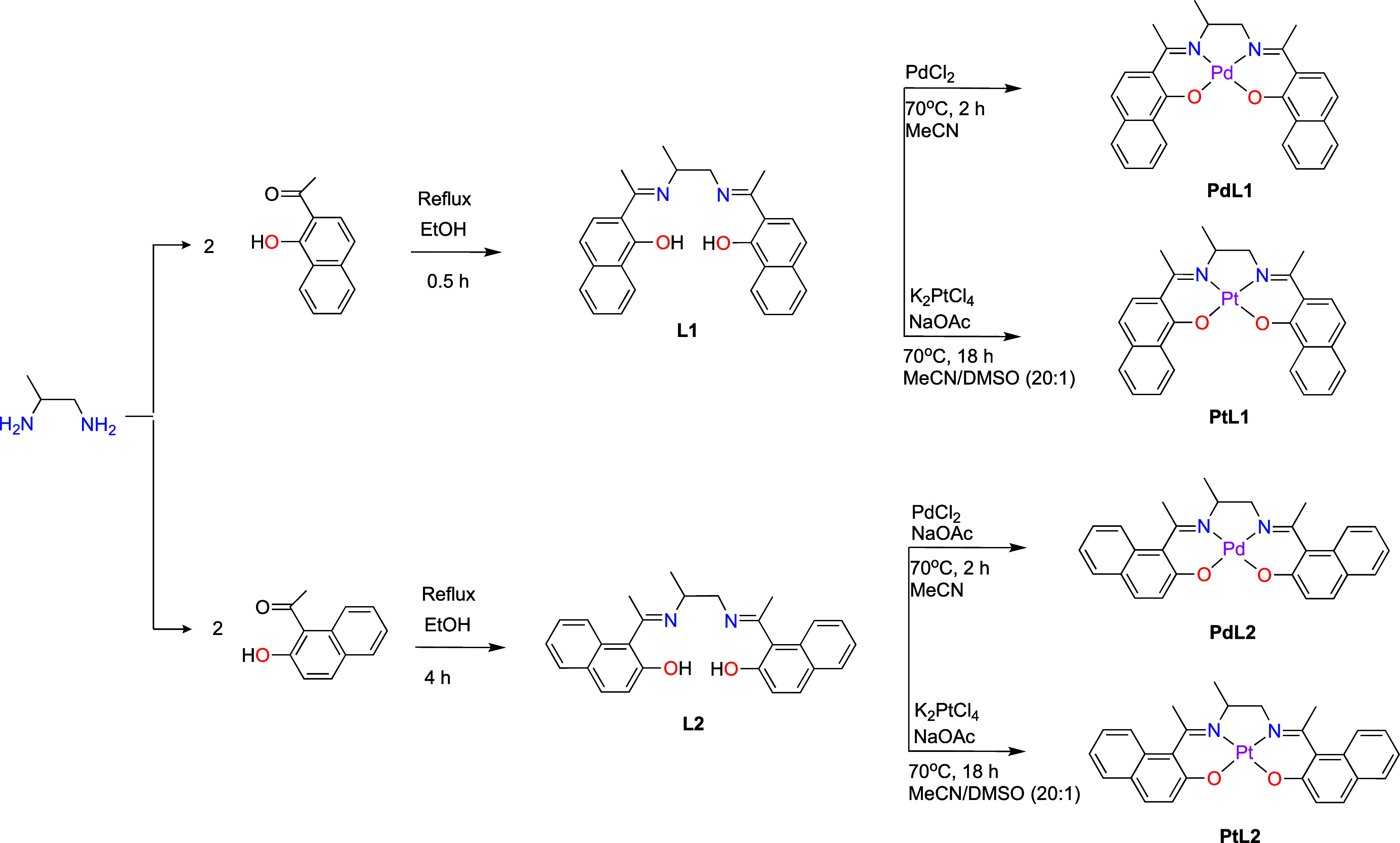
Synthetic Pathway to the Salen-Like Ligands L1 and L2 and the Corresponding Pt­(II) and Pd­(II) Complexes (PdL1, PdL2, PtL1, and PtL2)

### Characterization

The synthesized naphthalene-substituted salen ligands (**L1** and **L2**) and their corresponding Pd­(II) and Pt­(II) complexes (**PdL1**, **PdL2**, **PtL1**, and **PtL2**) were characterized using UV–vis spectroscopy, ^1^H NMR spectroscopy, single-crystal X-ray crystallography, and C, H, N elemental analysis.

### UV–vis Spectroscopy

The UV–vis spectra of the complexes **L1**, **L2**, **PdL1**, **PdL2**, **PtL1**, and **PtL2** were recorded in dichloromethane (DCM) at a concentration of 0.3 mM (Supporting Information Figure S2 and Table S1). Ligand **L1** exhibited three peaks at 325, 417, and 434 nm. The first absorption peak at 325 nm corresponds to a π–π* intraligand transition of the naphtholate chromophore, while the remaining two peaks are attributed to π–π* transitions involving the lone pair electrons on the azomethine bonds. Similarly, **L2** showed absorption peaks at wavelengths comparable to those of **L1**, with peaks at 325, 417, and 434 nm. Upon coordination with Pd­(II) and Pt­(II), the resulting complexes (**PdL1**, **PdL2**, **PtL1**, and **PtL2**) displayed absorption peaks analogous to those of the free ligands, indicating similar electronic transitions and successful metalation. For instance, **PdL1** exhibited peaks at 332, 408, and 431 nm, while **PdL2** showed peaks at 311, 414, and 436 nm. **PtL1** displayed peaks at 325, 434, and 448 nm, whereas **PtL2** had peaks at 321, 343, and 435 nm. Notably, the intensity of the π–π* transition associated with the naphtholate chromophores (310–335 nm for **L1** and 300–325 nm for **L2**) increased upon coordination, suggesting enhanced electronic delocalization facilitated by metal–ligand charge transfer (MLCT).^26,29^ In contrast, the transitions associated with the imine moiety (375–435 nm) exhibited reduced intensity upon coordination, likely due to the shorter M–N bond length, which reflects stronger metal–ligand interactions.^26,29^ Similar spectral features, including characteristic peaks, were observed in our previous studies for **L1** and **PtL1**.^26^

### ^1^H NMR Spectroscopy

The ^1^H NMR spectra confirmed the successful synthesis of the ligands and complexes. The characteristic signals for the methyl-substituted azomethine (–CH_3_–C=N-) protons in **L1** and **L2** appeared around δ 2–3 ppm and were slightly upfield shifted upon metal coordination. This shift indicates an increase in electron density around the protons, likely due to the electronic effects of the metal centers, further supporting successful complexation (Figure S3). Moreover, this upfield shift is consistent with a MLCT interaction, where electron density is partially transferred from the metal center to the ligand, affecting the local magnetic environment of nearby protons.

### Single-Crystal X-Ray Crystallography

Single crystals suitable for X-ray diffraction analysis were obtained by slow evaporation of a concentrated CHCl_3_ solutions. Three new crystal structures for the **PdL1**, **PdL2**, and **PtL2** complexes are reported herein, while the structure of **PtL1** was previously described in our earlier studies.^26^ The **PdL1**, **PdL2**, **PtL1**, and **PtL2** complexes crystallized in the monoclinic space groups *P*2_1_/*c*, *C*2/*c*, *P*2_1_/*c*, and *C*2/*c*, respectively, and all exhibit a square planar geometry around the central metal atom (Figure 2 and Table S2). The torsion angles for the O1–N1–N2–O2 and O1–N1–N2-M bonds are −0.43° and 0.42°, 4.30° and 2.61°, −0.24° and 0.16°, and −2.70° and −1.66°, respectively.^26^ These torsion angles provide insight into the planarity and geometric distortion of the metal complexes. Angles near 0° or ±180° indicate that the atoms lie within or close to the same plane, characteristic of ideal square planar geometries. This arrangement optimizes ligand coordination and minimizes structural strain. The minimal deviations observed in **PdL1** (−0.43° and 0.42°) and **PtL1** (−0.24° and 0.16°) suggest highly planar structures with negligible distortions. In contrast, slightly higher deviations in **PdL2** (4.30° and 2.61°) and **PtL2** (−2.70° and −1.66°) indicate mild geometric distortions.

**Figure 2:**
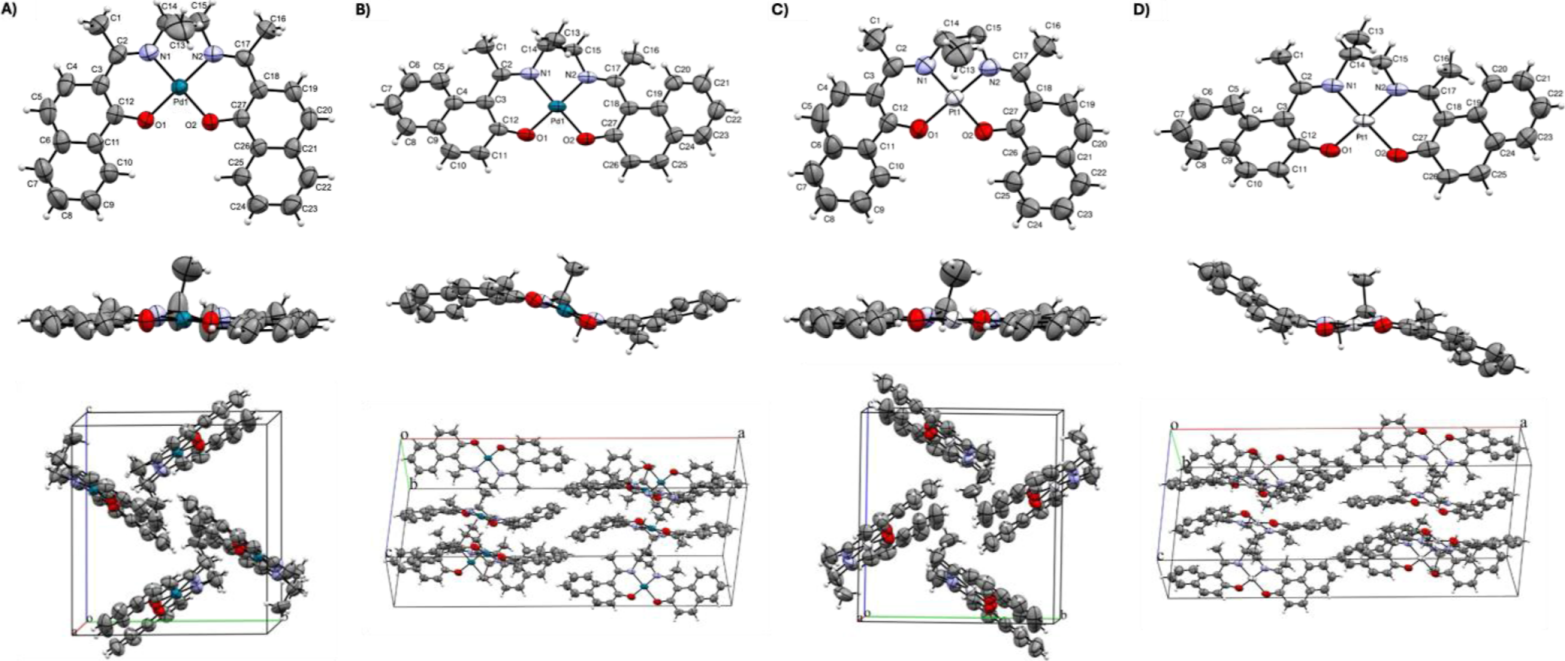
Crystal structures of metallosalens: (A) PdL1 (2128741), (B) PdL2 (2450474), (C) PtL1 (2129562),^26^ and (D) PtL2 (2450470), showing front, side, and packing views along the *b*-axis. The structures are depicted as ORTEP diagrams at 50% probability, illustrating the thermal displacement ellipsoids of the atoms.

The square planar geometry was further validated by calculating the τ_4_ and τ′_4_ parameters, which reflect the degree of distortion from planarity. Values close to zero indicate minimal deviation from the ideal geometry. Specifically, the τ_4_ and τ′_4_ parameters were determined as (τ_4_ = 0.013 and τ′_4_ = 0.011) for **PdL1**, (τ_4_ = 0.051 and τ′_4_ = 0.048) for **PdL2**, (τ_4_ = 0.008 and τ′_4_ = 0.007) for **PtL1**, and (τ_4_ = 0.036 and τ′_4_ = 0.035) for **PtL2** (Table S2). This square planar geometry, along with the minor distortions observed in Pd­(II) and Pt­(II) complexes, is consistent with structural features that may facilitate DNA intercalation or insertion.^9^ Despite the extended aromatic group in different orientations, the planar arrangement around the metal center is still favored, with values for the **L1** complexes closer to 0 than those for **L2**, yet both are in the 0.00–0.05 range. The planar arrangement around the metal center can enable effective π-stacking interactions with nucleobases, enhancing the potential for DNA binding.^9^ Furthermore, slight distortions in Pd­(II) and Pt­(II) complexes may contribute to flexibility in binding modes, potentially enhancing their affinity for DNA and influencing their biological activity.

### Elemental Analysis

The elemental analysis confirmed the composition and purity of the synthesized the ligands and complexes. Each compound exhibited a purity of >95%, determined by comparing the experimental (Found) and theoretical values of carbon (C), hydrogen (H), and nitrogen (N) content (Table S3).

### Biological Study

#### In Vitro Antiproliferative Activity against NCI 60 Cell-Lines Panel

The in vitro antiproliferative activity of ligands **L1** and **L2**, at a concentration of 10 μM, was evaluated using the National Cancer Institute’s (NCI) 60-cell-line panel, which includes nine types of cancer such as leukemia, nonsmall-cell lung cancer (NSCLC), colon cancer, central nervous system (CNS) cancer, melanoma, ovarian cancer, renal cancer, prostate cancer, and breast cancer. The results indicate that both ligands exhibited selective behavior depending on the cancer cell type, with significant variations in cell growth promotion (GP). For ligand **L1**, relatively low antiproliferative activity was observed in most leukemia cell lines, with the most affected being CCRF-CEM (GP = 88.62%) and RPMI-8226 (GP = 87.56%). However, in NSCLC, **L1** showed higher effectiveness, especially in the EKVX cell line, where significant inhibition of cell growth was observed (GP = 51.27%). Other NSCLC cell lines, such as HOP-62 and NCI–H522, exhibited greater resistance, with GP values of 95.51% and 97.95%, respectively. **L1** also demonstrated moderate activity in colon cancer, with the HCT-15 cell line reaching a GP of 88.85%. In CNS cancer cell lines, the activity was more varied, with SF-268 showing a GP of 88.07%, while SF-539 displayed greater resistance (GP = 97.84%). Melanoma cell lines were also resistant to **L1**, with SK-MEL-5 showing a GP of 97.71%. Renal and breast cancer cell lines exhibited moderate resistance to **L1**, with GPs ranging from 87.53% (UO-31) to 93.33% (T-47D). On the other hand, ligand **L2** exhibited somewhat broader antiproliferative activity, although still limited, with the leukemia cell line MOLT-4 being the most affected (GP = 92.95%). In NSCLC, HOP-92 was the most sensitive cell line to **L2**, with a GP of 86.51%. Similar to **L1**, other NSCLC cell lines, such as NCI–H522, showed moderate resistance (GP = 85.10%). In colon cancer, **L2** resulted in a GP of 91.60% for HCC-2998. CNS cancer cell lines were mostly resistant to **L2**, with SF-539 showing a GP of 96.88%. In melanoma, **L2** exhibited limited effectiveness, with LOX IMVI reaching a GP of 92.67%. In renal cancer, the CAKI-1 cell line showed moderate growth inhibition (GP = 85.49%), while other lines, such as 786–0, were more resistant (GP = 97.71%). Regarding breast cancer cell lines, **L2** demonstrated moderate activity, with T-47D standing out with a GP of 87.45%. These findings reflect the selective nature of the antiproliferative activity of ligands **L1** and **L2** across different cell lines in the NCI-60 panel, suggesting that structural variations in the ligands influence their interaction with cancer cells and, consequently, their cytotoxic potential. The results of the single-dose screen are given in the Supporting Information (Figures S4, S5).

#### Cytotoxic Activity against A375 and H292 Cancer Cell Lines and Healthy Lung Cell Line (HSAEC)

The cytotoxic activity of the **L1**, **L2**, **PdL1**, **PdL2**, **PtL1**, and **PtL2** complexes was evaluated against A375 (melanoma), H292 (nonsmall cell lung cancer), and HSAEC (healthy lung epithelial) cell lines to assess their selective cytotoxicity and potential as anticancer agents. The incucyte cytotox green assay was employed to monitor cell death in real-time over a 20 h period across six concentrations (0.5, 18, 36, 54, 72, and 90 μM) (Figure 3). **PtL1** and **PtL2** exhibited significant cytotoxicity in both A375 and H292 cell lines, as evidenced by increased green fluorescence indicative of membrane disruption (Figure 4). Notably, **PtL1** showed superior cytotoxicity in the A375 cell line compared to **PtL2**, suggesting that the directionality of the ligand in the **PtL1** complex may contribute to its enhanced activity. In contrast, no significant differences in cytotoxicity were observed between **PtL1** and **PtL2** in the H292 cell line, highlighting the influence of cell-specific interactions on the efficacy of these complexes. **L1**, **L2**, **PdL1**, and **PdL2** displayed limited activity under the same conditions. The notable fluorescence increase in **PtL1** and **PtL2** highlights their superior anticancer potential compared to their free ligands and Pd-containing analogs.

**Figure 3:**
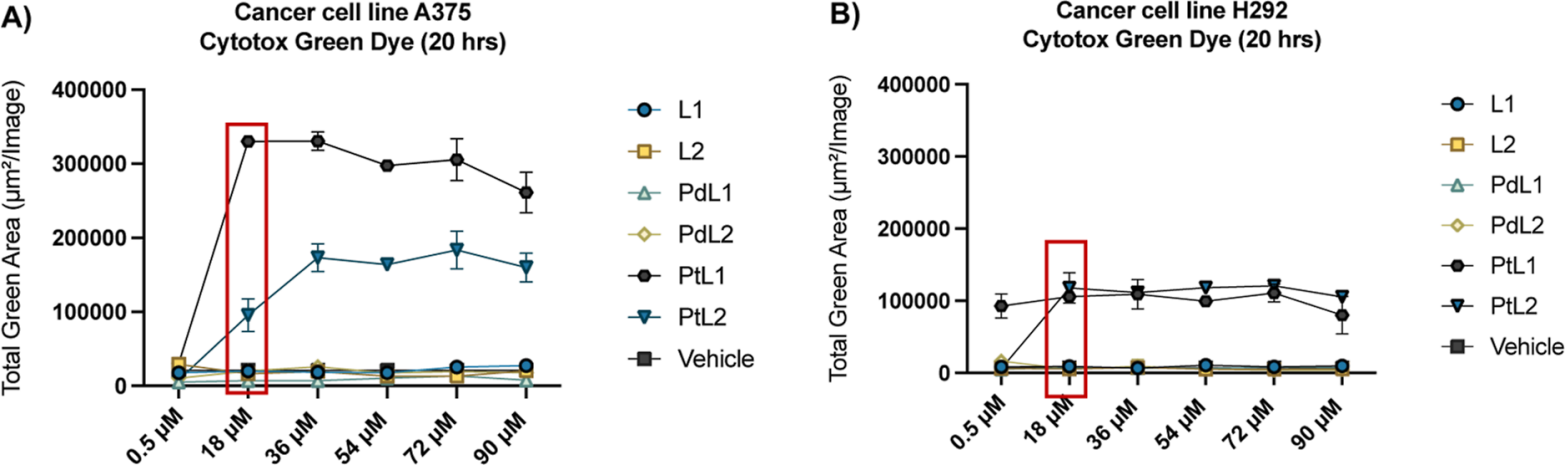
Cytotoxicity assessment using incucyte cytotox green. The graphs (A and B) illustrates the cytotoxicity induced by various treatments, including vehicle (DMSO 2% in media, negative control), L1, L2, PdL1, PdL2, PtL1, and PtL2 in the A375 (melanoma) and H292 (lung cancer) cell lines over a 20 h period at (0.5, 18, 36, 54, 72, 90) μM. Graphs represent the total green area, indicative of cell death, for each treatment at different concentration, expressed as mean ± SD. A red rectangle highlights the concentration of 18 μM, where the highest cytotoxicity was observed.

**Figure 4:**
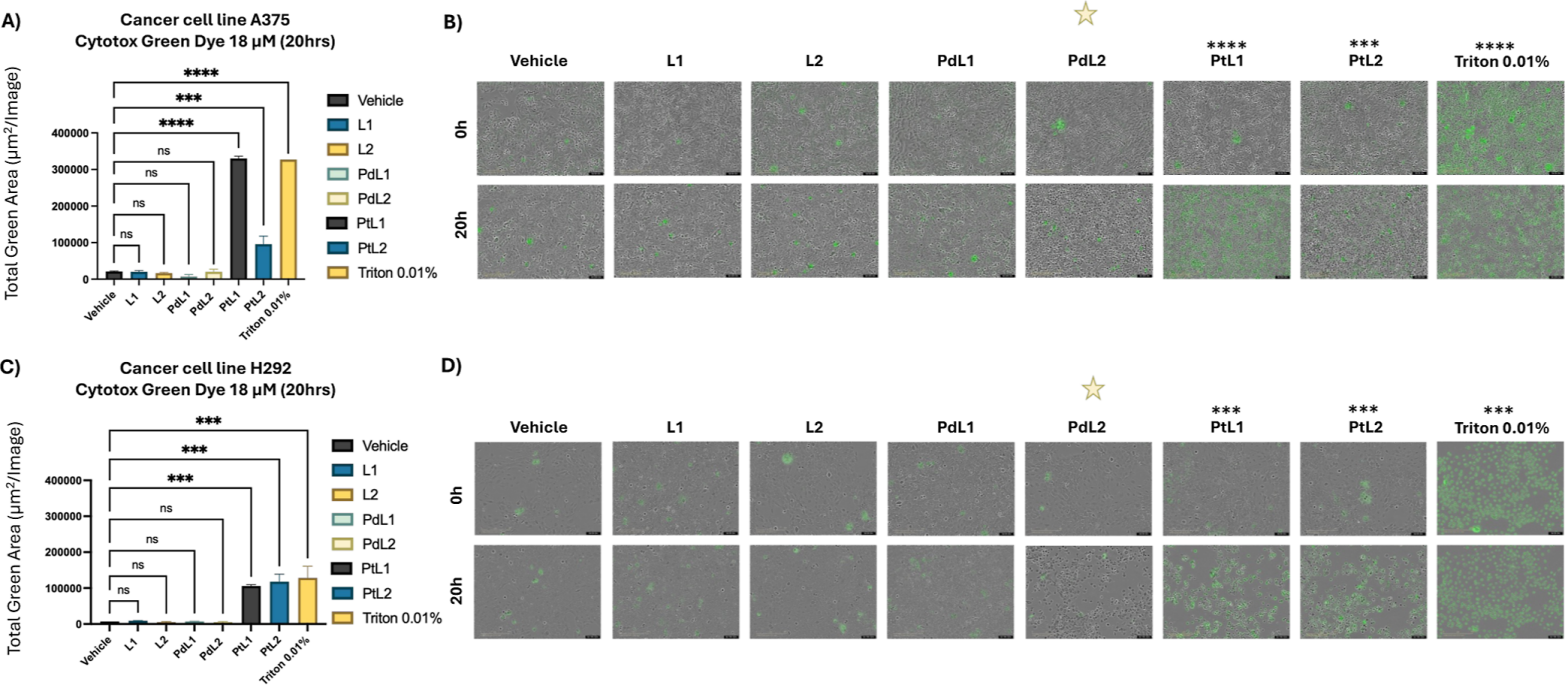
Cytotoxicity assessment using incucyte cytotox green against H292 and A375 cancer cell lines. This figure illustrates the cytotoxicity induced by various treatments, including vehicle (DMSO 2% in media, negative control), L1, L2, PdL1, PdL2, PtL1, PtL2, and Triton 0.01% (positive control), in the (A and B) A375 (melanoma) and (C and D) H292 (lung cancer) cell lines over a 20 h period at 18 μM. The graphs (A and C) represent the total green area, indicative of cell death, for each treatment, expressed as mean ± SD. Quantification of the total green area was achieved using Incucyte’s integrated image analysis tools, which capture fluorescence emitted by cells with compromised membranes, indicating cytotoxicity. Representative images (B and D) display the cells in real-time treatment at 0 and 20 h. A one-way ANOVA analysis of the total green area mean values revealed statistically significant differences between treatments, with *p*-values reported as 0.1234 (ns), 0.0332 (*), 0.0021 (**), 0.0002 (***), and <0.0001 (****). Yellow stars indicate regions of morphological changes without associated green fluorescence, characterized by a loss of cell adherence, suggesting structural alterations.

Real-time images were captured every hour throughout the 20 h period and compiled into videos (see Supporting Information, Figure S6). **PdL2** induced noticeable morphological changes in A375 and H292 cells, including loss of adherence and cell rounding. These findings suggest that **PdL2** disrupts cellular structure, potentially impairing adhesion-dependent processes. The ability of **PdL2** to reduce adhesion could be relevant in limiting metastasis, as “adhesion is the first step in the process of tumor cell invasion, and the formation and disassembly of adhesion drive the migration cycle.”^30^ These findings underline the importance of the ligand’s directional interactions in influencing the biological activity of these complexes.

To evaluate potential off-target effects, the cytotoxicity of the compounds was assessed in HSAEC cells. At both 18 μM and 90 μM (Figure S7), no statistically significant differences in green fluorescence were observed between treated samples and the control (vehicle). The mild fluorescence signal from the vehicle control (2% DMSO) likely reflects baseline cytotoxicity but did not obscure the results, confirming that the compounds do not harm healthy lung cells. These findings are crucial in demonstrating the selectivity of the tested compounds toward cancer cells. The enhanced cytotoxicity of **PtL1** and **PtL2** compared to **PdL1** and **PdL2** likely arises from the synergistic effects of the platinum center and the orientation of the naphthalene moiety within the ligand framework. While **PdL1** and **PtL1** share identical ligands, as are those in **PdL2** and **PtL2**, the presence of Pt­(II) appears to play a more significant role in enhancing cytotoxicity through stronger ligand–field interactions, greater electronic effects, and higher reactivity compared to Pd­(II). These properties may amplify critical interactions such as DNA binding and cellular uptake, which are critical for inducing cytotoxic effects. In addition, the altered orientation of the naphthalene moiety may enhance stacking interactions with DNA bases. Further computational studies are needed to confirm this hypothesis. Furthermore, **PtL1** and **PtL2** may exploit cancer cell-specific features more effectively due to the unique electronic properties of Pt­(II). Cancer cells with altered membrane compositions, increased fluidity, and upregulated transport proteins may facilitate the preferential uptake of these platinum-containing complexes, enhancing their efficacy in cancer cells while sparing healthy cells.^31,32^ In contrast, the lower reactivity and different electronic characteristics of Pd­(II) could limit the activity of **PdL1** and **PdL2**, despite their similar ligand frameworks.

#### Half-Maximal Inhibitory Concentration (IC_50_)

The cytotoxic activity of the metallosalens **PdL1**, **PdL2**, **PtL1**, and **PtL2**, along with the reference agents oxaliplatin and carboplatin, was evaluated in A375 and H292 cell lines over a 20 h period (Figure S8). The resulting IC_50_ values are summarized in Table S4. Pt­(II) complexes exhibited markedly higher cytotoxic activity than their Pd­(II) counterparts in both cell lines. Specifically, **PdL1** and **PdL2** showed no measurable IC_50_ values within the tested range (0.5–13.5 μM), indicating limited cytotoxicity under these conditions. In contrast, **PtL1** demonstrated the greatest potency, with IC_50_ values of 0.48 ± 0.07 μM in A375 and 0.83 ± 0.08 μM in H292. **PtL2**, although less potent than **PtL1**, remained cytotoxic, with IC_50_ values of 4.00 ± 1.24 μM (A375) and 4.19 ± 1.74 μM (H292). For comparison, oxaliplatin exhibited IC_50_ values of 7.80 ± 4.83 μM in A375 and >13.5 μM in H292, while carboplatin showed an IC_50_ of 10.65 ± 4.78 mM in H292 and no measurable effect in A375. These results highlight the superior potency of **PtL1**, not only relative to its Pd­(II) analogues but also when compared to oxaliplatin and carboplatin. The enhanced cytotoxicity of **PtL1** may be attributed in part to its highly planar geometry (τ_4_ = 0.008), which facilitates π–π stacking interactions with biological targets such as DNA. In comparison, **PtL2** exhibits a more distorted geometry (τ_4_ = 0.036), potentially reducing such interactions. The favorable geometry, combined with the electronic contribution of the naphthalene moiety, likely contributes to the enhanced anticancer activity of **PtL1**. These findings position **PtL1—**and to a lesser extent **PtL2—**as promising lead candidates for further development.

#### Cytotoxicity Compared to Chemotherapy Agent Oxaliplatin

The cytotoxic activity of the **PdL1**, **PdL2**, **PtL1**, and **PtL2** complexes was evaluated in comparison to the platinum-based drug oxaliplatin to assess their efficacy relative to a commercially available chemotherapeutic treatment. The incucyte cytotox green assay was used to quantify cytotoxic effects in A375 and H292 cell lines. Cells were exposed to five concentrations of the metallosalens (0.5, 1.1, 2.6, 5.9, and 13.5 μM) and oxaliplatin over a 20 h period (Figure 5). In A375 cells, **PdL2**, **PtL1**, **PtL2**, and oxaliplatin demonstrated significant cytotoxicity compared to the vehicle control (Figure 5A). Among these, **PtL1** induced the most pronounced effects, characterized by a substantial loss of cell adherence and distinctive morphological changes, surpassing the impact observed with oxaliplatin (Figure 6A,B). These results suggest that **PtL1** disrupts the cellular structure more effectively in A375 cells. Similarly, in H292 cells, **PdL2**, **PtL1**, and **PtL2** displayed significant cytotoxicity compared to oxaliplatin, as evidenced by increased green fluorescence (Figure 5B). **PtL1** and **PtL2** showed the highest fluorescence intensity and induced marked morphological changes, indicative of greater cytotoxic activity across all tested concentrations (Figure 6C,D). These findings indicate that **PtL1** and **PtL2** exert a stronger cytotoxic effect on both A375 and H292 cell lines compared to oxaliplatin. The prominent morphological changes and loss of adherence observed in cells treated with these complexes highlight their ability to disrupt cellular structure, supporting their potential as more effective alternatives to oxaliplatin in cancer treatment.

**Figure 5:**
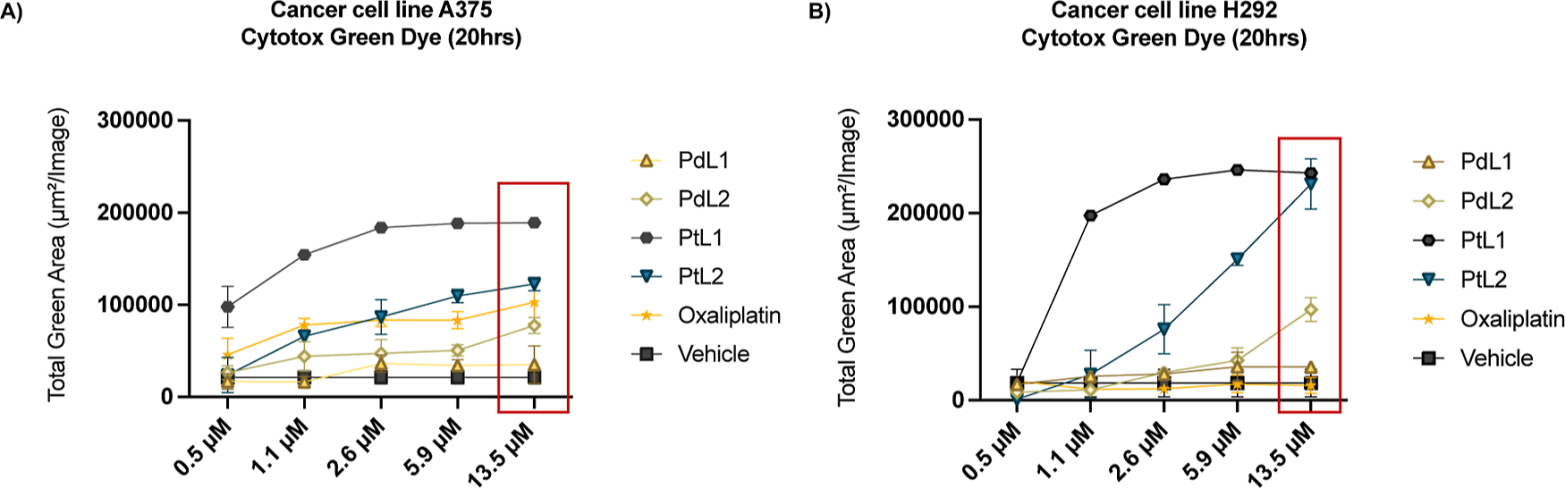
Cytotoxicity Comparison with Chemotherapy Agent Oxaliplatin. Graphs (A and B) show the cytotoxicity induced by various treatments, including vehicle (2% DMSO in media), PdL1, PdL2, PtL1, PtL2, and Oxaliplatin in the A375 and H292 cell lines over a 20 h period, with metallosalens tested at a 1:1 ratio to Oxaliplatin. These graphs represent the total green fluorescence area, indicating cell death, for each treatment at different concentrations, and are expressed as mean ± SD. A red rectangle highlights the 13.5 μM concentration, which will be further analyzed in bar graphs to compare the statistical significance between treatments in Figure 6.

**Figure 6:**
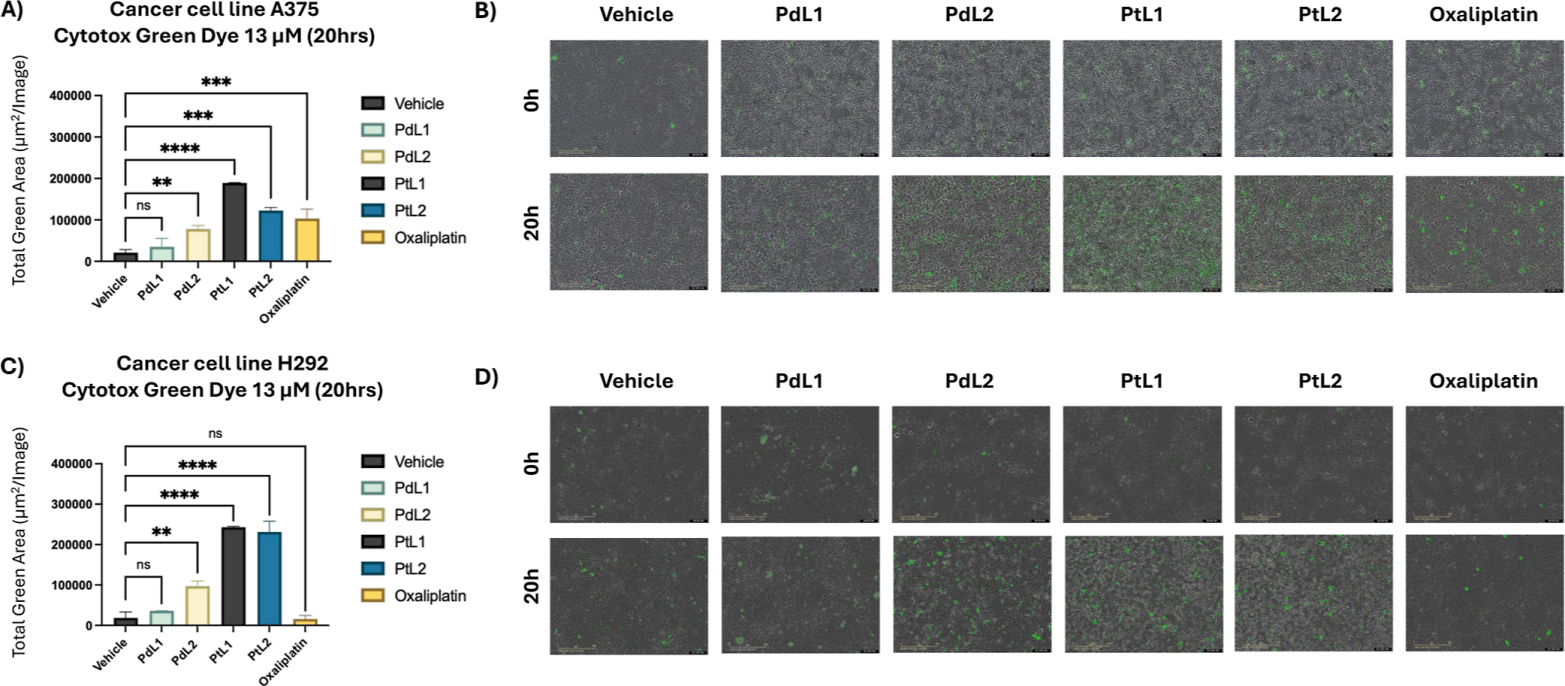
Cytotoxicity Comparison with Chemotherapy Agent Oxaliplatin in H292 and A375 Cancer Cell Lines. This figure illustrates the cytotoxicity effects of various treatments, including vehicle control (DMSO 2% in media) PdL1, PdL2, PtL1, PtL2, and Oxaliplatin, in the (A and B) A375 and (C and D) H292 cell lines at a concentration of 13.5 μM over 20 h. The graphs (A and C) show the total green area, representing cell death, measured as mean ± SD for each treatment. Quantification was performed using Incucyte’s integrated image analysis, which detects fluorescence from cells with compromised membranes, a marker of cytotoxicity. Representative images of treated cells at 0 and 20 h are shown in panels (B and D). A one-way ANOVA analysis of the total green area mean values revealed statistically significant differences between treatments, with *p*-values reported as 0.1234 (ns), 0.0332 (*), 0.0021 (**), 0.0002 (***), and <0.0001 (****).

#### Caspase 3/7 Green Dye

To evaluate the apoptotic execution in cancer cells treated with the synthesized compounds, the Caspase 3/7 Green Dye Assay was performed. A375 and H292 cell lines were exposed to 9 μM solutions of **L1**, **L2**, **PdL1**, **PdL2**, **PtL1**, and **PtL2** for 20 h (Figure 7). In the A375 cell line, treatments with **PtL1** and **PtL2** resulted in a significant increased Caspase 3/7 activation compared to the vehicle control, indicating a higher level of apoptosis (Figure 7A,B). Similarly, in the H292 cell line, significant apoptosis was observed for **PtL1**, **PtL2**, and **PdL2** treatments relative to the vehicle control (Figure 7C,D). These findings align with the results from the incucyte cytotox green assay, confirming that the cytotoxic effects previously observed were mediated through programmed cell death via apoptosis. Consistent findings have been reported in other studies, where metallosalens were shown to induce apoptosis and exhibit strong cytotoxicity against cultured cancer cells.^33–37^ Most importantly, the Pt metallosalens (**PtL1** and **PtL2**) under study and **PdL2** demonstrated selective induction of apoptosis in cancer cells, highlighting their strong potential as novel anticancer agents.

**Figure 7:**
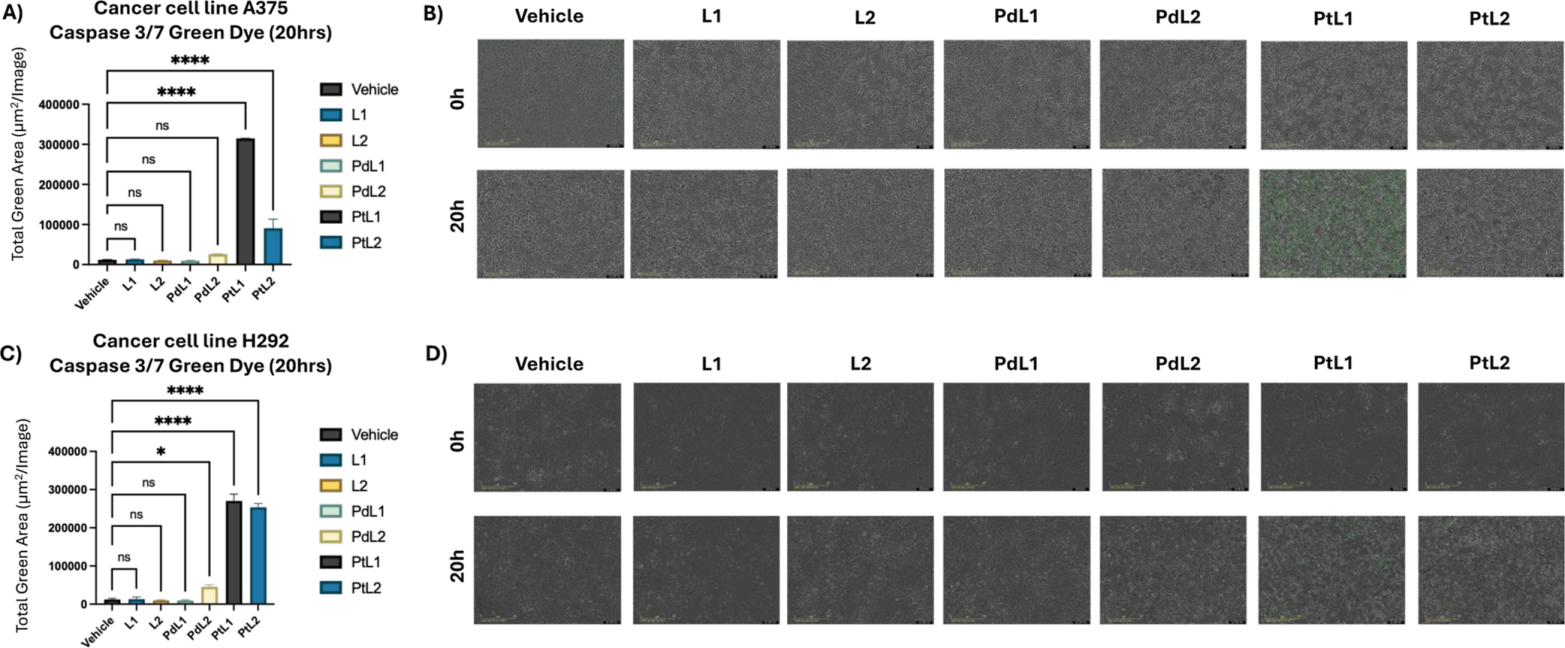
Caspase 3/7 Green Dye in H292 and A375 Cancer Cell Lines. This figure illustrates the effects of various treatments, including vehicle control (DMSO 2% in media), L1, L2, PdL1, PdL2, PtL1, and PtL2 in the (A and B) A375 and (C and D) H292 cell lines at a concentration of 9 μM over 20 h. The bar graphs (A and C) show the total green area, representing Caspase 3/7 activation, measured as mean ± SD for each treatment. The Caspase 3/7 Green Dye emits green fluorescence upon cleavage by activated caspases 3 and 7 during apoptosis. Quantification was performed using Incucyte’s integrated image analysis, which detects fluorescence from cells. Representative real-time images of treated cells at 0 and 20 h are shown in panels (B and D). A one-way ANOVA analysis of the total green area mean values revealed statistically significant differences between treatments, with *p*-values reported as 0.1234 (ns), 0.0332 (*), 0.0021 (**), 0.0002 (***), and <0.0001 (****).

## Conclusion

We synthesized and characterized a series of naphthalene-modified Pt­(II) and Pd­(II) metallosalen complexes with defined square planar geometries and minimal coordination site distortions, supporting their potential for π-stacking DNA interactions. Among them, **PtL1** and **PtL2** exhibited potent and selective cytotoxicity toward A375 (melanoma) and H292 (nonsmall cell lung cancer) cell lines, outperforming Pd analogues and oxaliplatin. **PtL1** showed enhanced activity in A375, while both **PtL1** and **PtL2** demonstrated comparable effects in H292, indicating that ligand orientation and cell-specific interactions influence biological performance. **PdL2** induced pronounced morphological changes, including loss of adhesion, suggestive of disruption of adhesion-dependent processes relevant to metastasis. All complexes demonstrated low cytotoxicity in healthy epithelial cells, and apoptosis was confirmed as the principal mechanism of action via caspase activation. Taken together, these results validate naphthalene-modified Pt­(II) metallosalens—particularly **PtL1** and **PtL2**—as promising anticancer candidates. Their performance underscores the importance of rational ligand and metal center design for achieving selective and mechanistically defined cytotoxic activity.

## Experimental Section

### General Procedures

All reactions were performed under an inert atmosphere using a Schlenk line. Solvents were dried using standard techniques and used as received unless otherwise specified. Reagents and solvents were sourced from CCSI (Toa Baja, Puerto Rico), Thermo Fisher Scientific (Cayey, Puerto Rico), and VWR (Manatí, Puerto Rico). The progress of all reactions was monitored on TLC using DCM/ethyl acetate (2:8) as solvent system. The precipitate was collected by filtration a washed with diethyl ether (Et_2_O) for **L1** and **L2**, or acetonitrile (MeCN) for metallosalens. The solids from all reactions were dried under vacuum and recrystallized in chloroform. UV–vis absorption measurements were recorded using a Shimadzu UV-1900 spectrophotometer (Kyoto, Japan) using DCM as solvent. Proton (^1^H) NMR spectra were obtained on a Bruker Ascend Aeon 500 MHz spectrometer (Faellanden, Switzerland) using CDCl_3_ and DMSO-*d*_6_ as solvents. Chemical shifts are reported in parts per million (ppm), relative to tetramethylsilane (TMS) as the internal standard. Single-crystal X-ray diffraction data were collected using a Rigaku SuperNova diffractometer equipped with a HyPix3000 X-ray detector and a Cu–Kα (λ = 1.5417 Å) radiation source (The Woodlands, TX, USA). The new crystal structures of **PdL1**, **PdL2**, and **PtL2** were deposited in the Cambridge Crystallographic Data Centre (CCDC) under deposition numbers 2128741, 2450474, and 2450470, respectively. These entries include detailed crystallographic data and can be accessed via the CCDC database. Elemental analysis was performed using vario EL cube CHNS elemental analyzer. The purity of all the compounds was determined to be >95%, by comparing the experimental (Found) and theoretical values of carbon (C), hydrogen (H), and nitrogen (N) content. In addition, the purity of the compounds used in biological assays (**L1**, **L2**, **PdL1**, **PdL2**, **PtL1**, and **PtL2**) was confirmed by analytical HPLC. The analyses were performed on a Waters 2690 system using a C18 reversed-phase column (4.6 × 150 mm, 5 μm) using a gradient of acetonitrile and water at a flow rate of 1.0 mL/min and column temperature of 25 °C. UV detection was carried out at compound-specific wavelengths optimized for each structure. All ligands and metallosalens used in biological and analytical studies showed HPLC purity ≥97%. Representative chromatograms are provided in the Supporting Information (Figure S9 and Table S5). This dual-method approach ensures that the compounds meet the purity requirements for biological evaluation through scientifically rigorous validation.

### General Procedure for the Synthesis of the Salen-Like Ligands and Characterization

The synthesis of **L1** (2′-((1′,1′E)-(propane-1,2-diylbis­(azaneylylidene))­bis­(ethan-1-yl-1-ylidene))­bis­(naphthalen-1-ol)) was carried out following the procedure described in our previous work.^25,26^ Specifically, 1-hydroxy-2-acetonaphthone (Sigma-Aldrich, CAS No. 711-79-5) (2 equiv, 1.4074 g) was added to a three-neck round-bottom flask along with 20 mL of ethanol (EtOH) and stirred until fully dissolved. Subsequently, 1,2-diaminopropane (Sigma-Aldrich, CAS No. 78-90-0) (1 equiv, 0.35 mL) was carefully added to the reaction mixture using a syringe. The reaction mixture was refluxed for 30 min, during which a yellow solid precipitated. The resulting solid was isolated by filtration, washed several times with Et_2_O, dried under vacuum, and recrystallized in chloroform. Yield: 87%. ^1^H NMR (500 MHz, CDCl_3_, δ): ppm: 1.53–1.54 (3H, d, C=N–C­(H)–CH_3_, *J* = 6.45 Hz), 2.33 (3H, s, N=C–CH_3_), 2.41 (3H, s, N=C–CH_3_), 3.74–3.85 (2H, m, –CH_2_, *J* = 9.58 Hz), 4.30 (1H, m, –CH), 6.81–6.84 (2H, dd, Ar–H, *J*_1_ = 5.38 Hz, *J*_2_ = 5.37 Hz), 7.21–7.24 (2H, t, Ar–H, *J* = 17.25 Hz), 7.40–7.42 (2H, t, Ar–H, *J* = 15.06 Hz), 7.49–7.52 (2H, t, Ar–H, *J* = 7.42 Hz), 7.58–7.59 (2H, d, Ar–H, *J* = 7.83 Hz), 8.45–8.46 (2H, d, Ar–H, *J* = 8.18 Hz). UV–vis (DCM, λ_max_, nm [ε, M^–1^ cm^–1^]): 325 [7167], 417 [12800], 434 [12400]. **L1** crystallizes in the monoclinic space group *P*2_1_/*n*.^29^ Elemental analysis calculated (%) for C_27_H_26_N_2_O_2_: C 79.00, H 6.38, N 6.82. Found: C 77.85, H 6.18, N 6.68. Purity: 97.79%. **L1**: retention time = 11.762 min; peak area, >99%; eluent A,H_2_O; eluent B, MeCN; linear gradient over 20 min at 1 mL min^–1^; detection at 269 nm; column temperature, 25 °C.

For the synthesis of **L2** (1′-((1′,1′E)-(propane-1,2-diylbis­(azaneylylidene))­bis­(ethan-1-yl-1-ylidene))­bis­(naphthalen-2-ol)), 2-hydroxy-1-acetonaphthone (TCI, CAS No. 574-19-6) (1.5214 g, 2 equiv) was dissolved in ethanol (20 mL) in a three-neck round-bottom flask. To this solution, 1,2-diaminopropane (0.35 mL, 1 equiv) was added dropwise using a syringe. The reaction mixture was refluxed for 4 h, during which a yellow solid precipitated. The solid was filtered, washed with diethyl ether (Et_2_O), and dried under vacuum. Yield: 83%. ^1^H NMR (500 MHz, DMSO-*d*_6_, δ): ppm: 1.49–1.50 (3H, d, C=N–C­(H)–CH_3_, *J* = 6.40 Hz), 2.55 (3H, s, N=C–CH_3_), 3.36–3.40 (3H, m, N=C–CH_3_, *J* = 7.00 Hz), 3.98–3.99 (2H, t, –CH_2_, *J* = 5.23 Hz), 4.50–4.55 (1H, m, –CH, *J* = 6.53 Hz), 6.76–6.80 (2H, t, Ar–H, *J* = 9.53 Hz), 7.34–7.38 (2H, m, Ar–H, *J* = 6.90 Hz), 7.43–7.45 (2H, dd, Ar–H, *J*_1_ = 3.53 Hz, *J*_2_ = 3.28 Hz), 7.50–7.53 (2H, t, Ar–H, *J* = 6.93 Hz), 7.61–7.64 (2H, t, Ar–H, *J* = 6.34 Hz), 8.29–8.31 (2H, dd, Ar–H, *J*_1_ = 3.78 Hz, *J*_2_ = 3.82 Hz). UV–vis (DCM, λ_max_, nm [ε, M^–1^ cm^–1^]): 325 [5917], 417 [10583], 434 [10283]. Elemental analysis calculated (%) for C_27_H_26_N_2_O_2_: C 79.00, H 6.38, N 6.82. Found: C 75.84, H 6.13, N 6.55. Purity: 96.04%. **L2**: retention time = 9.706 min; peak area, >99%; eluent A,H_2_O; eluent B, MeCN; linear gradient over 20 min at 1 mL min^–1^; detection at 247 nm; column temperature, 25 °C.

### General Procedure for the Synthesis of the Pd (II) Metallosalens and Characterization

**PdL1** (2′-((1′,1′E)-(propane-1,2-diylbis­(azaneylylidene))­bis­(ethan-1-yl-1-ylidene))­bis­(naphthalen-1-ol)-κ^4^N,N′,O,O′-palldium-(II)). Ligand **L1** (1 equiv, 0.0800 g) was dissolved in 10 mL of MeCN in a three-neck round-bottom flask. The solution was stirred at 70 °C until complete dissolution. PdCl_2_ (STREM, CAS No.7647-10-1) (1 equiv, 0.0346 g) dissolved in 5 mL of MeCN was added dropwise to the solution. The reaction mixture was stirred and heated at 70 °C for 2 h. The resulting solid was isolated by filtration, washed several times with MeCN, dried under vacuum, and recrystallized in chloroform. Yield: 63%. Notably, no base was added to assist ligand deprotonation during this synthesis. In contrast to the procedures used for other complexes, the absence of NaOAc may have contributed to the lower yield of PdL1 by reducing the efficiency of metal coordination. ^1^H NMR (500 MHz, CDCl_3_, δ): ppm: 1.25 (3H, m, C=N–C­(H)–CH_3_), 1.95 (3H, s, N=C–CH_3_), 2.01 (3H, s, N=C–CH_3_), 3.41–3.43 (1H, d, –CH_2_, *J* = 13.13 Hz), 3.83–3.84 (1H, m, –CH_2,_ *J* = 4.87 Hz), 4.31–4.33 (1H, d, –CH_,_ *J* = 13.08 Hz), 6.79–6.82 (1H, dd, Ar–H, *J*_1_ = 4.04 Hz, *J*_2_ = 4.38 Hz), 6.91–6.94 (1H, dd, Ar–H *J*_1_ = 4.13 Hz, *J*_2_ = 4.71 Hz), 7.12–7.13 (1H, d, Ar–H, *J* = 4.66 Hz), 7.27–7.30 (1H, dd, Ar–H, *J*_1_ = 4.24 Hz, *J*_2_ = 5.05 Hz), 7.52–7.51 (4H, d, Ar–H, *J* = 9.33 Hz), 7.62–7.60 (2H, d, Ar–H, *J* = 9.39 Hz), 8.85 (2H, s, Ar–H). UV–vis (DCM, λ_max_, nm [ε, M^–1^ cm^–1^]): 332 [19967], 408 [11933], 431 [12933]. **PdL1** crystallizes in the monoclinic space group *P*2_1_/*n* (Table S2). Elemental analysis calculated (%) for C_27_H_24_N_2_O_2_Pd: C 62.98, H 4.70, N 5.44. Found: C 61.50, H 4.55, N 5.35. Purity: 97.60%. **PdL1**: retention time = 14.083 min; peak area, >99%; eluent A, H_2_O; eluent B, MeCN; linear gradient over 20 min at 1 mL min^–1^; detection at 286 nm; column temperature, 25 °C.

**PdL2** (1′-((1′,1′E)-(propane-1,2-diylbis­(azaneylylidene))­bis­(ethan-1-yl-1-ylidene))­bis­(naphthalen-2-ol)-κ^4^N,N′,O,O′-palladium­(II)). Ligand **L2** (1 equiv, 0.0800 g) was dissolved in 10 mL of MeCN in a three-neck round-bottom flask. The solution was stirred at 70 °C until completely dissolved. Afterward, 2 equiv of sodium acetate (NaOAc) were suspended in the **L2** solution. Then, a 5 mL solution of PdCl_2_ (1 equiv, 0.0346 g) in MeCN was added to the mixture. The reaction was stirred and heated at 70 °C for 2 h. The resulting solid was isolated by filtration, washed several times with MeCN, dried under vacuum, and recrystallized in chloroform. Yield of 95%. ^1^H NMR (500 MHz, CDCl_3_, δ): ppm: 1.57–1.58 (3H, d, C=N–C­(H)–CH_3_, *J* = 5.87 Hz), 2.58 (3H, s, N=C–CH_3_), 2.62 (3H, s, N=C–CH_3_), 3.52–3.55 (1H, d, –CH_2_, *J* = 13.24 Hz), 3.98–4.02 (1H, dd, –CH_2_, *J*_1_ = 4.51 Hz, *J*_2_ = 4.57 Hz), 4.18–4.22 (1H, m, –CH, *J* = 11.40 Hz), 7.15–7.19 (2H, m, Ar–H, *J* = 19.45 Hz), 7.29–7.34 (4H, m, Ar–H, *J* = 8.02 Hz), 7.47–7.51 (2H, dd, Ar–H, *J*_1_ = 8.45 Hz, *J*_2_ = 8.47 Hz), 7.52–7.56 (2H, t, Ar–H, *J* = 8.63 Hz), 7.61–7.64 (2H, t, Ar–H, *J* = 11.65 Hz). UV–vis (DCM, λ_max_, nm [ε, M^–1^ cm^–1^]): 311 [28567], 414 [9000], 436 [8300]. **PdL2** crystallizes in the point group *C*2/*c* (Table S2). Elemental analysis calculated (%) for C_27_H_24_N_2_O_2_Pd: C 62.98, H 4.70, N 5.44. Found: C 61.20, H 4.50, N 5.25. Purity: 96.13%. **PdL2**: retention time = 12.714 min; peak area, >99%; eluent A,H_2_O; eluent B, MeCN; linear gradient over 20 min at 1 mL min^–1^; detection at 269 nm; column temperature, 25 °C.

### General Procedure for the Synthesis of the Pt (II) Metallosalens and Characterization

**PtL1** (2′-((1′,1′E)-(propane-1,2-diylbis­(azaneylylidene))­bis­(ethan-1-yl-1-ylidene))­bis­(naphthalen-1-ol)-κ^4^N,N′,O,O′-platinum­(II)). Ligand **L1** (1 equiv, 0.1700 g) was dissolved in 20 mL of MeCN in a three-neck round-bottom flask. The solution was stirred at 70 °C until completely dissolved. Afterward, 2 equiv of NaOAc were suspended in the **L1** solution. Then, a 1 mL solution of K_2_PtCl_4_ (Sigma-Aldrich, CAS No. 10025-99-7) (1 equiv, predissolved in dimethyl sulfoxide, DMSO) was added to the reaction mixture. The reaction was stirred and heated at 70 °C for 8 h under an inert atmosphere. The resulting solid was isolated by filtration, washed several times with MeCN, dried, and recrystallized in chloroform. Yield of 95%. ^1^H NMR (500 MHz, CDCl_3_, δ): ppm: 1.28–1.31 (3H, d, C=N–C­(H)–CH_3_, *J* = 6.62 Hz), 1.91 (3H, s, N=C–CH_3_), 1.97 (3H, s, N=C–CH_3_), 3.46–3.49 (1H, d, –CH_2_, *J* = 12.95 Hz), 3.88–3.91 (1H, m, –CH_2_, *J* = 12.17 Hz), 4.23–4.27 (1H, dd, –CH, *J*_1_ = 5.30 Hz, *J*_2_ = 5.22 Hz), 6.91–6.93 (1H, d, Ar–H, *J* = 9.12 Hz), 7.00–7.02 (1H, d, Ar–H, *J* = 9.14 Hz), 7.32–7.34 (1H, d, Ar–H, *J* = 9.12 Hz), 7.45–7.47 (1H, d, Ar–H, *J* = 9.14 Hz), 7.53–7.57 (2H, m, Ar–H, *J* = 7.14 Hz), 7.62–7.69 (4H, m, Ar–H, *J* = 9.03 Hz), 8.95–8.97 (2H, d, Ar–H, *J* = 8.22 Hz). UV–vis (DCM, λ_max_, nm [ε, M^–1^ cm^–1^]): 325 [20967], 434 [5467], 448 [4867]. **PtL1** crystallizes in the monoclinic *P*2_1_/*c* space group with a square planar geometry for the metal center (Table S2).^26^ Elemental analysis calculated (%) for C_27_H_24_N_2_O_2_Pt: C 53.73, H 4.01, N 4.64. Found: C 52.75, H 3.95, N 4.56. Purity: 98.31%. **PtL1**: retention time = 14.019 min; peak area, 97%; eluent A, H_2_O; eluent B, MeCN; linear gradient over 20 min at 1 mL min^–1^; detection at 246 nm; column temperature, 25 °C.

**PtL2** (1′-((1′,1′E)-(propane-1,2-diylbis­(azanelylidene))­bis­(ethan-1-yl-1-ylidene))­bis­(naphthalen-2-ol)-κ^4^N,N′,O,O′-platinum­(II)). Ligand **L2** (1 equiv, 0.1700 g) was dissolved in 20 mL of MeCN in a three-neck round-bottom flask. The solution was stirred at 70 °C until completely dissolved. Afterward, 2 equiv of NaOAc were suspended in the L2 solution. Then, a 1 mL solution of K_2_PtCl_4_ (1 equiv, predissolved in dimethyl sulfoxide, DMSO) was added to the reaction mixture. The reaction was stirred and heated at 70 °C for 8 h under an inert atmosphere. The resulting solid was isolated by filtration, washed several times with diethyl ether (Et_2_O), and dried under vacuum, and recrystallized in chloroform. Yield: 93%. ^1^H NMR (500 MHz, CDCl_3_, δ): ppm: 1.56–1.57 (3H, d, C=N–C­(H)–CH_3_, *J* = 6.42 Hz), 2.40 (3H, s, N=C–CH_3_), 2.45 (3H, s, N=C–CH_3_), 3.56–3.59 (1H, d, –CH_2_, *J* = 12.91 Hz), 4.07–4.10 (1H, dd, –CH_2_ *J*_1_ = 4.78 Hz, *J*_2_ = 4.81 Hz), 4.16–4.21 (1H, m, –CH *J* = 6.02 Hz), 7.20–7.24 (2H, dd, Ar–H, *J*_1_ = 7.23 Hz, *J*_2_ = 7.11 Hz), 7.31–7.40 (4H, m, Ar–H, *J* = 8.75 Hz), 7.51–7.58 (2H, dd, Ar–H, *J*_1_ = 8.44 Hz, *J*_2_ = 8.45 Hz), 7.65–7.71 (4H, m, Ar–H, *J* = 5.78 Hz). UV–vis (DCM, λ_max_, nm [ε, M^–1^ cm^–1^]): 321 [23600], 343 [14633], 435 [5867]. **PtL2** crystallizes in the monoclinic *P*2_1_/*c* space group with a square planar geometry for the metal center (Table S2). Elemental analysis calculated (%) for C_27_H_24_N_2_O_2_Pt: C 53.73, H 4.01, N 4.64. Found: C 52.55, H 3.90, N 4.50. Purity: 97.36%. **PtL2**: retention time = 12.843 min; peak area, >99%; eluent A, H_2_O; eluent B, MeCN; linear gradient over 20 min at 1 mL min^–1^; detection at 277 nm; column temperature, 25 °C.

#### In Vitro Anti-Proliferative Activity against the NCI-60 Cell Line Panel

The salen-like ligands were selected by the National Cancer Institute (NCI), NIH, USA, under the Developmental Therapeutics Program (DTP) for the evaluation of their in vitro antiproliferative activity against the NCI-60 cell line panel. This screening platform includes human tumor cell lines representing melanoma, leukemia, colon, lung, ovarian, brain, prostate, kidney, and breast cancers. The NCI screening service assesses compounds with potential drug-like mechanisms of action using computer-aided design tools. A key criterion for selection is the ability of compounds to contribute to the chemical diversity of the NCI small molecule compound collection. The salen-like ligands were assigned NCI codes 846214 (**L1**) and 847889 (**L2**), representing the chemotype developed in this study. The ligands were evaluated in an initial one-dose (10 μM)% inhibition assay across the full NCI-60 cell line panel.

#### Cytotoxic Activity against H292, A375, and HSAEC Cell Lines

The A375 (melanoma) (ATCC, Cat. No. CRL-1619), H292 (NSCLC) (ATCC, Cat. No. CRL-1848), and HSAEC (healthy lung epithelial) (ATCC, Cat. No. PCS-301-010) cell lines were chosen for their biological relevance and to assess the selectivity and cytotoxicity of the novel metallosalen complexes. H292 cells represent a widely used model for nonsmall cell lung cancer, the leading cause of cancer-related mortality globally,^6,38^ while A375 cells are a well-established model for melanoma, a cancer strongly linked to UV exposure and of increasing prevalence in high-sunlight regions such as Puerto Rico.^39^ HSAEC cells were included as a noncancerous control to evaluate the differential cytotoxicity of the complexes in healthy tissue. The Incucyte SX5 Live-Cell Analysis System and the Incucyte Cytotox Green reagent (Sartorius, Cat. No. 9500–4633), a cyanine nucleic acid dye that enables real-time evaluation and quantification of cell death, were used (Sartorius, Cytotox Green Protocol). This assay was performed to assess cell viability and morphological changes induced by the compounds. The incubation conditions were consistent throughout all steps. H292, A375, and HSAEC cells with >95% confluence (grown in standard tissue culture flasks, Corning, Cat. No. CLS3290) were separately seeded into 96-well plates (Thermo Fisher, Cat. No. 167008) in a volume of 150 μL at a concentration of 3 × 10^4^ cells/mL. RPMI 1640 medium (Cytiva, Cat No. SH30255.01) supplemented with 10% fetal bovine serum (FBS) (Sigma-Aldrich, Cat. No. F0926) was used for the H292 cell line, while DMEM medium (Sigma-Aldrich, Cat. No. D6429) supplemented with 10% FBS (Thermo Fisher, Cat. No. A5670701) and 1% penicillin–streptomycin (P/S) (Gibco, Cat. No. 15140122) was used for the A375 cells. For the HSAEC cell line, Airway Epithelial Cell Basal Medium (ATCC, Cat. No. PCS-300-030) was supplemented with the Bronchial Epithelial Cell Growth Kit (ATCC, Cat. No. PCS-300-040) to prepare a complete medium. A volume of 150 μL per well was used at a concentration of 3 × 10^4^ cells/mL. Cells were allowed to attach for 24 h at 37 °C in a humidified air atmosphere with 5% CO_2_. Following this procedure, cancer cells were treated with different compounds at concentrations of 0.5, 18, 36, 54, 72, and 90 μM, along with the Incucyte Cytotox Green reagent (final concentration: 250 nM). HSAEC were treated with different compounds at concentrations of 18 and 90 μM, along with same dye concentration. Solutions of the compounds were prepared using the respective media, and the old media from the cells were removed and replaced with fresh media containing the Cytotox Green dye. The plates were placed in the Incucyte SX5 Live-Cell Analysis System, and four pictures per well were taken every hour over a 20 h period. For fluorescence analysis, the software applied surface fit background correction with a fluorescence threshold of 2.0 Green Calibrated Units (GCU). The software automatically analyzed the images and calculated the total integrated green intensity (GCU × μm^2^/image) per well to quantify DNA release associated with cytotoxicity.

### Half-Maximal Inhibitory Concentration (IC_50_)

Cytotoxicity of metallosalens **PdL1**, **PdL2**, **PtL1**, and **PtL2**, as well as the reference drugs oxaliplatin (APOTEX, NDC 60505-6132-07) and carboplatin (bpiLabs, NDC 54288-164-01), was evaluated in A375 and H292 cell lines using the Incucyte SX5 Live-Cell Analysis System (Sartorius) and Cytotox Green Dye (Cat. No. 4633, Sartorius). Cells were seeded in 96-well plates at a density of 3 × 10^4^ cells/mL in 150 μL per well and incubated for 24 h to allow for attachment. Subsequently, cells were treated with serial dilutions of the test compounds: 0.5, 1.1, 2.6, 5.9, and 13.5 μM for metallosalens and oxaliplatin; 0.5, 1.1, 2.5, 5.9, and 13.5 mM for carboplatin. Cytotox Green Dye was added to a final concentration of 250 nM to detect loss of membrane integrity. Plates were incubated for 20 h, and images were acquired and analyzed using Incucyte software. Cytotoxicity was quantified based on total green fluorescence intensity (green calibrated units, GCU × μm^2^/image), reflecting DNA release. IC_50_ values were calculated using nonlinear regression with a four-parameter dose–response model in GraphPad Prism (v10). All experiments were conducted in triplicate, and data are reported as mean ± standard deviation. All handling of oxaliplatin and carboplatin, classified as cytotoxic chemotherapeutic agents, was performed exclusively within a certified biological safety cabinet to ensure safe preparation and dilution. Standard laboratory safety procedures were followed, including the use of personal protective equipment such as gloves, lab coats, and protective eyewear. All waste generated was disposed of in accordance with institutional protocols for hazardous materials.

#### Cytotoxicity Compared to Chemotherapy Agent Oxaliplatin

Cytotoxicity was assessed over a 20 h time course in an Incucyte SX5 Live-Cell Analysis System incubator, as previously directed (Sartorius, Cytotox Green Protocol) using Incucyte Cytotox Green Dye (4633) for detecting cytotoxic disruption of cell membrane integrity. The same conditions for cell culture were maintained, where H292 and A375 cells were seeded at 3 × 10^4^ cells per well into 96-well plates and incubated for 24 h to allow attachment. Cells were treated with varying concentrations of salen-ligands (**L1** and **L2**), metallosalens (**PdL1**, **PdL2**, **PtL1** and **PtL2**), and oxaliplatin. Stock of oxaliplatin (APOTEX, NDC No. 60505-6132-07) used was 100 mg/20 mL in an Aqueous Solution and dilutions of 0.5, 1.1, 2.6, 5.9, and 13.5 μM were performed using cell medium 0.3% DMSO as solvent. Compound solutions of 0.5, 1.1, 2.6, 5.9, and 13.5 μM were done in the corresponding medium for each cell line. Old media from the cells was replaced by new media containing the Cytotox Green dye and compounds solutions were added to cells. Plates were placed in the Incucyte SX5 Live-Cell Analysis System, where pictures were taken hourly over 20 h. To ensure safe handling of oxaliplatin, a cytotoxic chemotherapeutic agent, all procedures involving its preparation and dilution were carried out exclusively within a biological safety cabinet. Standard safety protocols were followed, including the use of personal protective equipment such as gloves, lab coats, and protective eyewear. Waste materials were disposed of according to institutional guidelines for hazardous substances. The software processed the images and measured the total integrated green intensity (GCU × μm^2^/image) for each well to assess DNA release linked to cytotoxicity.

### Caspase 3/7 Green Dye

The activity of caspase 3 and caspase 7, key proteases typically activated during the apoptosis phase, was quantified using the Caspase 3/7 Green Dye (Sartorius, Cat. No. 4440). This assay is designed to detect the caspase-3 activation process in living cells by image analysis (Sartorius, Caspase-3/7 Green Protocol). Using this technique provides an insight with regards to the cell death mechanism that the treated cells are undergoing. The cell culture procedure was consistent with previous experiments. H292 and A375 cell lines were seeded at a density of 3 × 10^4^ cells per well into 96-well plates and incubated for 24 h at 37 °C in a humidified atmosphere containing 5% CO_2_. Following this, both H292 and A375 cell lines were treated with 9 μM solutions of each compound (**L1**, **L2**, **PdL1**, **PdL2**, **PtL1** and **PtL2**). The old cell media were replaced with fresh media containing the Caspase 3/7 Green Dye (final dye concentration: 5 μM), and the compound solutions were added to the wells. To monitor the process by image analysis, plates were placed in the Incucyte SX5 Live-Cell Analysis System for a 20 h period, where pictures were taken every hour. For fluorescence analysis, the software applied surface fit background correction with a fluorescence threshold of 2.0 Green Calibrated Units (GCU). The software automatically analyzed the images and calculated the total integrated green intensity (GCU × μm^2^/image) per well to quantify Caspase 3/7.

#### Statistics

Statistical analyses were performed using GraphPad Prism software. A one-way ANOVA was applied to analyze the mean values of the total green area, comparing the effects of different treatments. Statistically significant differences between treatments were determined, with *p*-values reported as follows: as 0.1234 (ns), 0.0332 (*), 0.0021 (**), 0.0002 (***), and <0.0001 (****).

## Supplementary Material





## Notes

Figure S1. Structure of Salen-like ligands. Figure S2. UV–vis spectrum in DCM of (A) L1, PdL1 and PtL2 (B) L2, PdL2 and PtL2. Table S1. Lambda max (λ_max_) and molar absorptivity coefficient (ε) of all compounds. Figure S3. ^1^H NMR spectra recorded in CDCl3 for (A) L1, (C) PdL1, (D) PdL2, (E) PtL1 and (F) PtL2, and in DMSO-*d*_6_ for (B) L2. Table S2. Crystallographic data of PdL1, PdL2, PtL125 and PtL2. Table S3. Elemental Analysis and Final Purity of L1, L2, PdL1, PdL2, PtL1, and PtL2. Figure S4. One dose bar graph (%) and mean graph form NCI 60 cancer cell line viability screen of L1 at the one-dose level for 48 h. Figure S5. One dose growth (%) and mean graph form NCI 60 cancer cell line viability screen of L2 at the one-dose level for 48 h. Figure S6. Real-time Imaging of A375 and H292 Cells Treated with 18 μM of Each Compound. Figure S7. Cytotoxicity Assessment Using Incucyte Cytotox Green Against HSAEC. Figure S8 Dose–response evaluation of cytotoxic activity in A) A375 and B) H292 cells treated with metallosalen complexes and standard chemotherapeutics. Table S4. IC_50_ values (μM or mM) of metallosalens and standard chemotherapeutic agents oxaliplatin and carboplatin in A375 and H292 cell lines. Figure S9. Analytical HPLC chromatograms of all ligands and metal complexes used in biological assays. Table S5 (PDF)
HPLC Retention Times and Purity Percentages of L1, L2, PdL1, PdL2, PtL1, and PtL2. Molecular Formula Strings (CSV)

## Abbreviations

A375: human melanoma cell line
BSC: biological safety cabinet
C: carbon
CCDC: Cambridge Crystallographic Data Centre
CNS: cancer nervous system
DCM: dichloromethane
DMEM: Dulbecco’s modified eagle medium
DMSO: dimethyl sulfoxide
DNA: DNA
Et_2_O: diethyl ether
FBS: fetal bovine serum
GP: growth promotion
H: hydrogen
H292: human nonsmall cell lung cancer cell line
HPLC: High-Performance Liquid Chromatography
HSAEC: human small airway epithelial cells
IC_50_: Inhibitory Concentration 50%
K_2_PtCl_4_: potassium tetrachloroplatinate­(II)
MLCT: metal-to-ligand charge transfer
N: nitrogen
NCI: National Cancer Institute
NSCLC: nonsmall cell lung cancer
NMR: nuclear magnetic resonance
O: oxygen
PdCl_2_: palladium­(II) chloride
P/S: penicillin/streptomycin
RPMI 1640: Roswell Park Memorial Institute 1640 medium
TLC: thin layer chromatography
TMS: tetramethylsilane
MeCN: acetonitrile
