# Supplementary material for: Evaluating Naphthalene-Modified Metallosalen Complexes as Anticancer Agents
Source: J Med Chem. 2025 Jul 15;68(14):14300–11. doi: 10.1021/acs.jmedchem.4c03180 (PMC12305649; doi:10.1021/acs.jmedchem.4c03180)
Supplement: Supplementary file 1 [file jm4c03180_si_001.pdf]

Supporting Information

Evaluating naphthalene-modified Metallosalen complexes as anticancer agents

Jemily Acosta-Mercado,[*,†,‡] Angelica Oliveras-Alsina,[†,‡] Ariana I. Marcano-Maiz,[†,‡] Andrea P. Rivera-Torres,[§] Keysha T. Cordero-Gimenez,[†,‡] Sonya Malavez-Cajigas,[§] Marvin J. Bayro,[†,‡] Marcos J. Ramos-Benitez,[§] Dalice M. Piñero Cruz[*,†,‡]

[†]Deparment of Chemistry, University of Puerto Rico, Rio Piedras Campus, 17 Ave Universidad STE 1701, San Juan, Puerto Rico 00925-2537, United States.

[‡]Molecular Science Research Center, Juan Ponce de León STE 1390, San Juan, Puerto Rico 00926, United States.

[§]Department of Basic Sciences, Ponce Health Sciences University, Ponce, Puerto Rico 00716, United States.

*E-mail: dalice.pinero@upr.edu (D.M.P.C.) and jemily.acosta@upr.edu (J.A.-M)

Table of Contents **Page**

Structure of Salen-like ligands…………………………………………………………………… S2

UV-Vis………………………………………………………………………………………. S3-S4

^{1}H-NMR………………………………………………………………………………… S5

Crystallographic data………………………………………………………………………… S6

Elemental Analysis…………………………………………………………………………… S7

NCI 60 cancer cell screening……………………………………………………………….. S8-S9

Real-time Imaging of A375 and H292 Cells Treatment…………………………………... S10

Cytotoxicity Assessment Against HSAEC…………………………………………………. S11

Half-Maximal Inhibitory Concentration IC_{50}…………………………………………………... S12-S13

Analytical HPLC Data…………………………………………………………………….... S14-S15

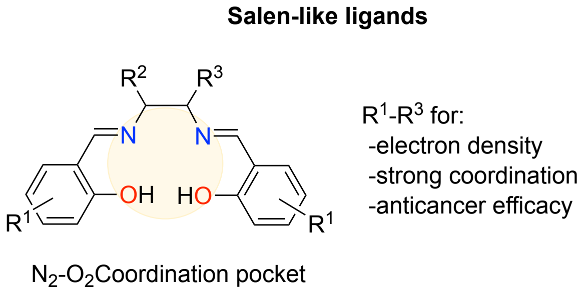

Figure S1. Structure of Salen-like ligands.

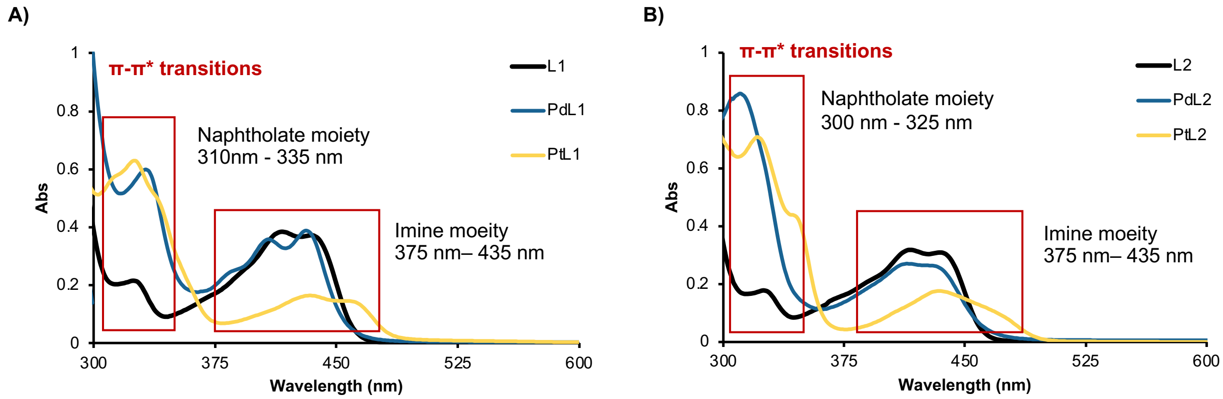

Figure S2. UV-Vis spectrum in DCM of (A) **L1**, **PdL1** and **PtL2** (B) **L2**, **PdL2** and **PtL2.** This figure illustrates the absorption profiles of the ligands (**L1** and **L2**) alongside their corresponding metal complexes (**PdL1**, **PtL1**, **PdL2**, and **PtL2**). The spectral shifts observed highlight the influence of metal coordination on the electronic transitions, reflecting the changes in the ligand's electronic environment upon complexation.

Compound	λmax (nm)	ε (dm3 mol-1cm-1)
L1	325	7167
	417	12800
	434	12400
L2	325	5917
	417	10583
	434	10283
PdL1	332	19967
	408	11933
	431	12933
PdL2	311	28567
	414	9000
	436	8300
PtL1	325	20967
	434	5467
	448	4867
PtL2	321	23600
	343	14633
	435	5867

Table S1. Lambda max (λmax) and molar absorptivity coefficient (ε) of all compounds.

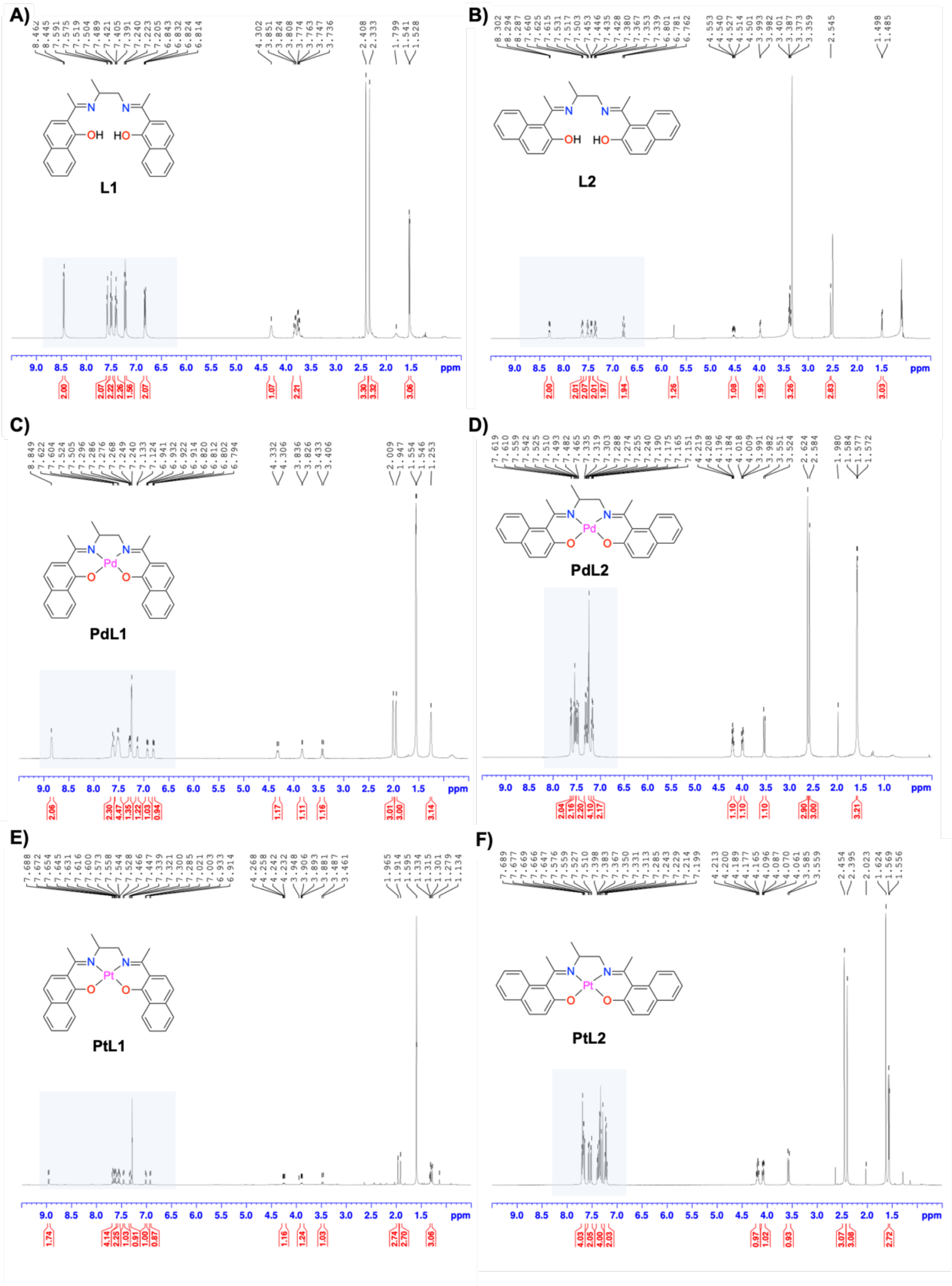

Figure S3. ^{1}H-NMR spectra recorded in $CDCl_3$ for (A) **L1**, (C) **PdL1**, (D) **PdL2**, (E) **PtL1** and (F) **PtL2**, and in DMSO-d_6 for (B) **L2**. *Note: The phenolic –OH signal is not observed in L1 (A), likely due to rapid exchange in $CDCl_3$. In L2 (B), a signal appears at δ 5.75 with an integration of 1.26 H instead of the expected 2 H, consistent with exchangeable phenolic protons. The difference is attributed to solvent effects: DMSO-d_6, being more polar and a better hydrogen bond acceptor, slows the exchange enough to partially observe the OH signal, while in $CDCl_3$ it is likely too fast to detect. This behavior is typical for Schiff base ligands and also reflects possible tautomeric equilibrium between enol–imine and keto–amine forms.*

	PdL1	PdL2	PtL1 (ref 26)	PtL2
**Empirical formula**	$C_{27}H_{24}N_2O_2Pd$	$C_{27}H_{24}N_2O_2Pd$	$C_{27}H_{24}N_2O_2Pt$	$C_{27}H_{24}N_2O_2Pt$
**Formula weight**	514.88	514.88	603.57	603.56
**Crystal Color**	orange	yellow	yellow	orange
**Crystal size (mm^3)**	0.2 × 0.14 × 0.09	0.131 × 0.097 × 0.047	0.14 × 0.08 × 0.04	0.067 × 0.052 × 0.040
**Crystal system**	monoclinic	monoclinic	monoclinic	monoclinic
**Space group**	$P2_1/c$	C2/c	$P2_1/c$	C2/c
**Temperature (K)**	300(10)	294.26(10)	293(2)	292.8(3)
**a (Å)**	13.76608(9)	32.9912(5)	13.74289(8)	32.9606(9)
**b (Å)**	12.20126(10)	10.35202(14)	12.23957(9)	10.3694(2)
**c (Å)**	13.11076(8)	13.03477(16)	13.11559(9)	13.0335(2)
**α (°)**	90	90	90	90
**β (°)**	99.0433(6)	94.3897(12)	98.9538(6)	93.924(2)
**γ (°)**	90	90	90	90
**Volume ($Å^3$)**	2174.76(3)	4438.65(11)	2179.25(3)	4444.17(16)
**Z**	4	8	4	8
**ρcalc (g/cm^3)**	1.573	1.541	1.840	1.804
**μ (mm^{-1})**	7.099	6.957	12.261	12.024
**Reflections collected**	40293	11857	49243	10907
**F(000)**	1048.0	2096.0	1176.0	2352.0
**Radiation**	Cu Kα (λ = 1.54184)	Cu Kα (λ = 1.54184)	Cu Kα (λ = 1.54184)	Cu Kα (λ = 1.54184)
**2Θ range for data collection/°**	6.502 to 136.696	5.374 to 136.768	6.51 to 136.81	5.374 to 136.778
**Index ranges**	-15 ≤ h ≤ 16, -14 ≤ k ≤ 14, -15 ≤ l ≤ 15	-39 ≤ h ≤ 39, -12 ≤ k ≤ 12, -11 ≤ l ≤ 15	-16 ≤ h ≤ 16, -14 ≤ k ≤ 14, -15 ≤ l ≤ 15	-39 ≤ h ≤ 35, -11 ≤ k ≤ 12, -9 ≤ l ≤ 15
**Independent reflections**	3995 [R_{int} = 0.0289, R_{sigma} = 0.0120]	4080 [R_{int} = 0.0252, R_{sigma} = 0.0285]	4008 [R_{int} = 0.0460, R_{sigma} = 0.0178]	4078 [R_{int} = 0.0382, R_{sigma} = 0.0439]
**Data/restraints/parameters**	3995/0/310	4080/0/293	4008/0/310	4078/0/385
**Goodness-of-fit on F^2**	1.067	1.057	0.985	1.001
**Final R indexes [I>=2σ(I)]**	R_1 = 0.0278, wR_2 = 0.0767	R_1 = 0.0258, wR_2 = 0.0649	R1 = 0.0228, wR2 = 0.0651	R_1 = 0.0283, wR_2 = 0.0577
**Final Rindexes [all data]**	R_1 = 0.0294, wR_2 = 0.0780	R_1 = 0.0307, wR_2 = 0.0672	R_1 = 0.0250, wR_2 = 0.0669	R_1 = 0.0420, wR_2 = 0.0627
**Largest diff. peak/hole / e $Å^{-3}$**	0.29/-0.68	0.81/-0.37	0.58/-0.91	0.47/-0.61
**Torsion angles O1-N1-N2-O2**	-0.43	4.30	−0.24	-2.70
**Torsion angles O1-N1-N2-M**	0.42	2.61	0.16	-1.66
**τ4 and τ'4**	0.013 and 0.011	0.051 and 0.048	0.008 and 0.007	0.036 and 0.035

Table S2. Crystallographic data of **PdL1**, **PdL2**, **PtL1**[26] and **PtL2**. The table includes key parameters such as formula weight, crystal size, space group, unit cell dimensions (a, b, c, and α, β, and γ), volume, density (ρcalc), reflection statistics, radiation source, data/parameter/restraints, final R indexes (R1, wR2), and torsion angles (O1-N1-N2-M). The data confirm the square planar geometry of the complexes with minimal distortions in the ligand coordination environment.

Compound	Formula	C (%) Theoretical	C (%) Found	H (%) Theoretical	H (%) Found	N (%) Theoretical	N (%) Found	Purity (%)
L1	$C_{27}H_{26}N_2O_2$	79.00	77.85	6.38	6.18	6.82	6.68	97.79
L2	$C_{27}H_{26}N_2O_2$	79.00	75.84	6.38	6.13	6.82	6.55	96.04
PdL1	$C_{27}H_{24}N_2O_2Pd$	62.98	61.50	4.70	4.55	5.44	5.35	97.60
PdL2	$C_{27}H_{24}N_2O_2Pd$	62.98	61.20	4.70	4.50	5.44	5.25	96.13
PtL1	$C_{27}H_{24}N_2O_2Pt$	53.73	52.75	4.01	3.95	4.64	4.56	98.31
PtL2	$C_{27}H_{24}N_2O_2Pt$	53.73	52.55	4.01	3.90	4.64	4.50	97.36

Table S3. Elemental Analysis and Final Purity of **L1**, **L2**, **PdL1**, **PdL2**, **PtL1**, and **PtL2.** The table shows the elemental analysis (C, H, N) and final purity of the ligands L1, L2, and their corresponding metal complexes (PdL1, PdL2, PtL1, PtL2). Purity was determined by combustion analysis.

Developmental Therapeutics Program

One Dose Bar Graph

NSC: D-846214 / 1 | **Conc:** 1.00E-5 Molar | **Test Date:** Jul 10, 2023

Experiment ID: 2307OS44 | **Report Date:** Oct 11, 2023

Panel/Cell Line	Growth Percent
Leukemia	
CCRF-CEM	88.62
HL-60(TB)	99.68
K-562	107.20
MOLT-4	114.46
RPMI-8226	87.56
SR	94.47
Non-Small Cell Lung Cancer	
A549/ATCC	101.85
EKVX	51.27
HOP-62	95.51
NCI-H226	105.96
NCI-H23	97.95
NCI-H322M	111.04
NCI-H460	107.11
NCI-H522	92.47
Colon Cancer	
COLO 205	111.04
HCC-2998	106.65
HCT-116	101.78
HCT-15	88.85
HT29	107.62
KM12	110.36
SW-620	101.41
CNS Cancer	
SF-268	88.97
SF-295	95.15
SF-539	97.84
SNB-75	85.63
U251	109.98
Melanoma	
LOX IMVI	91.49
MALME-3M	103.39
M14	111.79
MDA-MB-435	99.59
SK-MEL-2	104.14
SK-MEL-28	113.29
SK-MEL-5	97.71
UACC-257	108.65
UACC-62	98.41
Ovarian Cancer	
IGROV1	109.32
OVCAR-3	103.67
OVCAR-4	100.47
OVCAR-5	103.61
OVCAR-8	99.44
NCI/ADR-RES	106.31
SK-OV-3	111.02
Renal Cancer	
786-0	92.14
A498	105.88
ACHN	113.10
CAKI-1	91.71
RXF 393	101.04
SN12C	102.81
UO-31	87.53
Prostate Cancer	
PC-3	103.87
DU-145	105.69
Breast Cancer	
MCF7	88.15
MDA-MB-231/ATCC	99.45
HS 578T	101.31
BT-549	108.65
T-47D	93.33
MDA-MB-468	111.89

Bar Graph

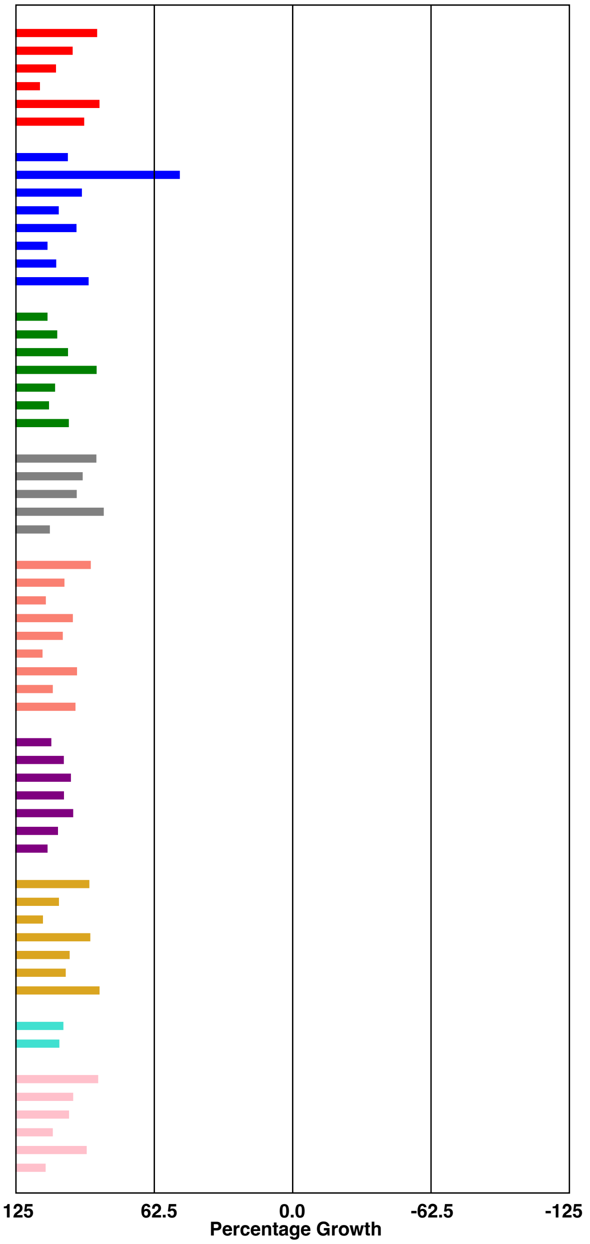

Figure S4. One dose bar graph (%) and mean graph form NCI 60 cancer cell line viability screen of **L1** at the one-dose level for 48 h.

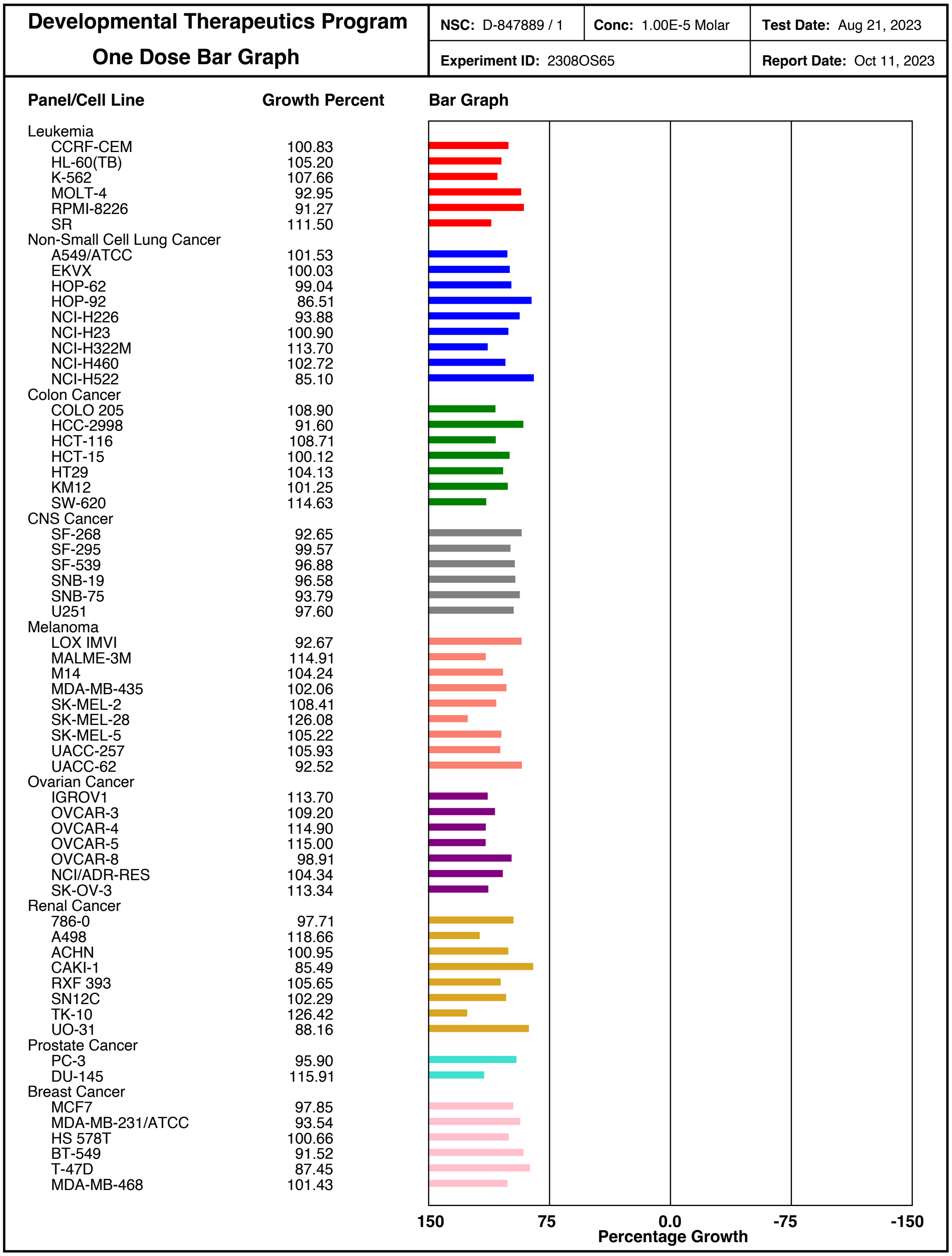

Figure S5. One dose growth (%) and mean graph form NCI 60 cancer cell line viability screen of **L2** at the one-dose level for 48 h.

A375

(a) L1

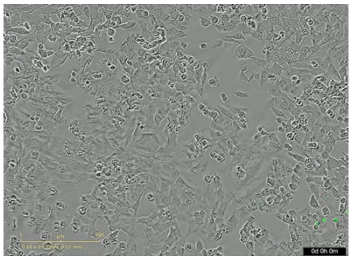

(c) PdL1

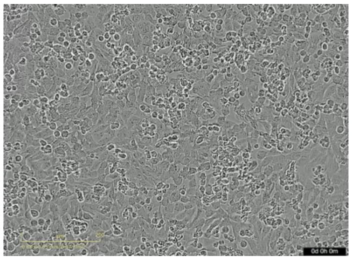

(e) PtL1

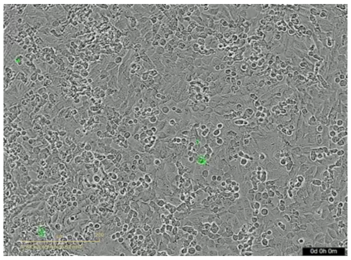

(b) L2

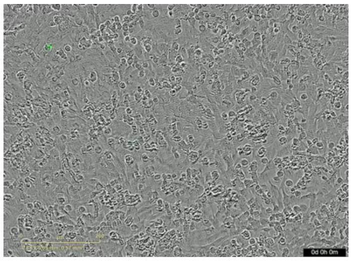

(d) PdL2

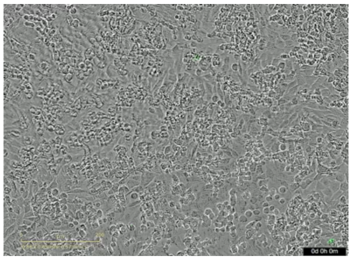

(f) PtL2

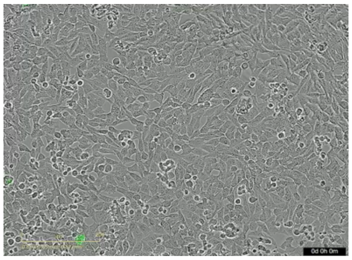

H292

(a) L1

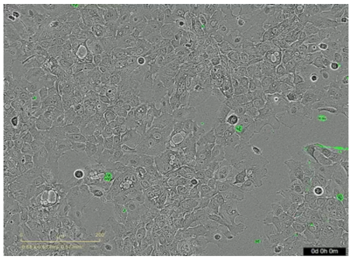

(c) PdL1

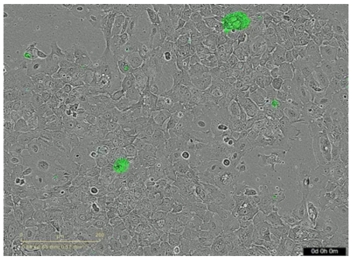

(e) PtL1

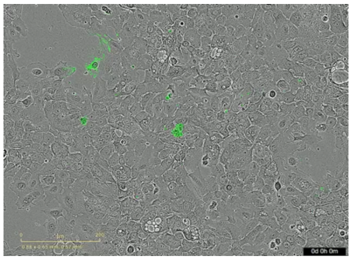

(b) L2

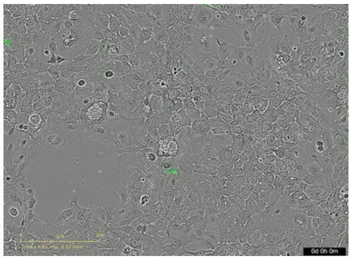

(d) PdL2

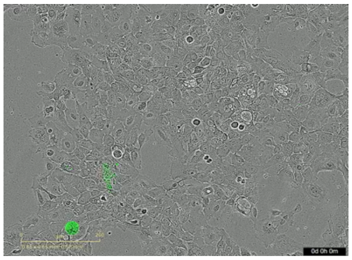

(f) PtL2

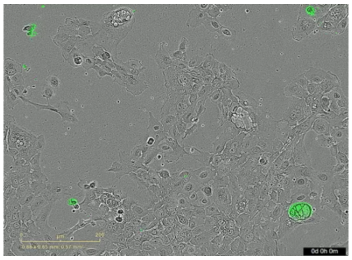

Figure S6. Real-time Imaging of A375 and H292 Cells Treated with 18 μM of Each Compound. Panels (a) through (f) display A375 and H292 cells exposed to (a) **L1**, (b) **L2**, (c) **PdL1**, (d) **PdL2**, (e) **PtL1**, and (f) **PtL2** over a 20-hour period, showing the progression of cell death. Images were captured every hour and compiled into a time-lapse video, with playback accelerated to 10x speed. Results indicate that **PtL1** and **PtL2** exhibit significant cytotoxic activity in both cancer cell lines, as evidenced by increased green fluorescence. In contrast, **L1**, **L2**, and **PdL1** show no significant cytotoxic effects. **PdL2**, however, leads to cell adherence loss, suggesting that this compound disrupts cellular structure.

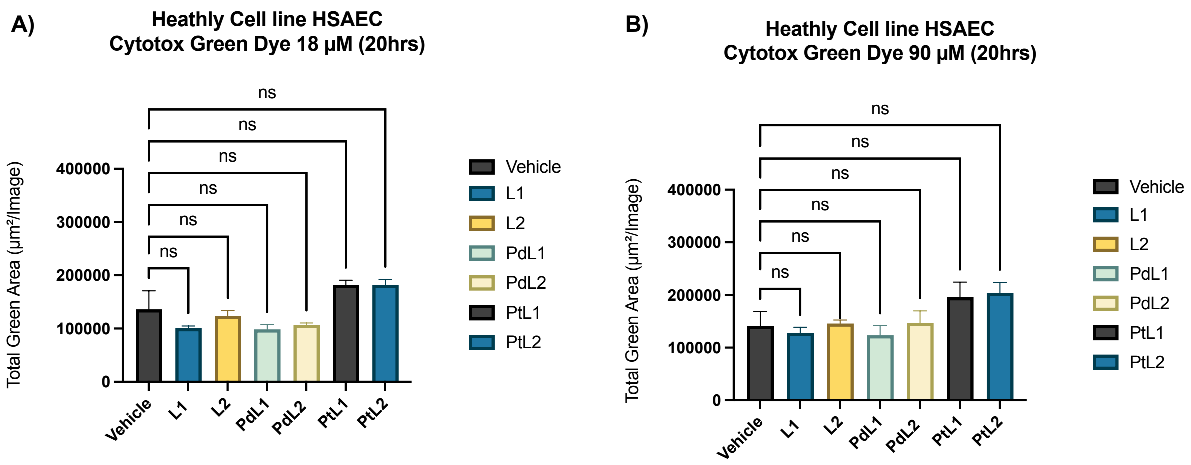

Figure S7. Cytotoxicity Assessment Using Incucyte Cytotox Green Against HSAEC. This figure illustrates the cytotoxicity induced by various treatments, including vehicle (DMSO 2% in media), **L1**, **L2**, **PdL1**, **PdL2**, **PtL1** and **PtL2**, in cell lines HSAEC cell line over a 20-hour period at (A) 18 µM and (B) 90 µM. The graphs (A and B) represent the total green area, indicative of cell death, for each treatment, expressed as mean ± SD. Quantification of the total green area was achieved using Incucyte's integrated image analysis tools, which capture fluorescence emitted by cells with compromised membranes, indicating cytotoxicity. *A one-way ANOVA analysis of the total green area mean values revealed statistically significant differences between treatments, with p-values reported as 0.1234 (ns), 0.0332 (*), 0.0021 (**), 0.0002 (***), and <0.0001 (****).*

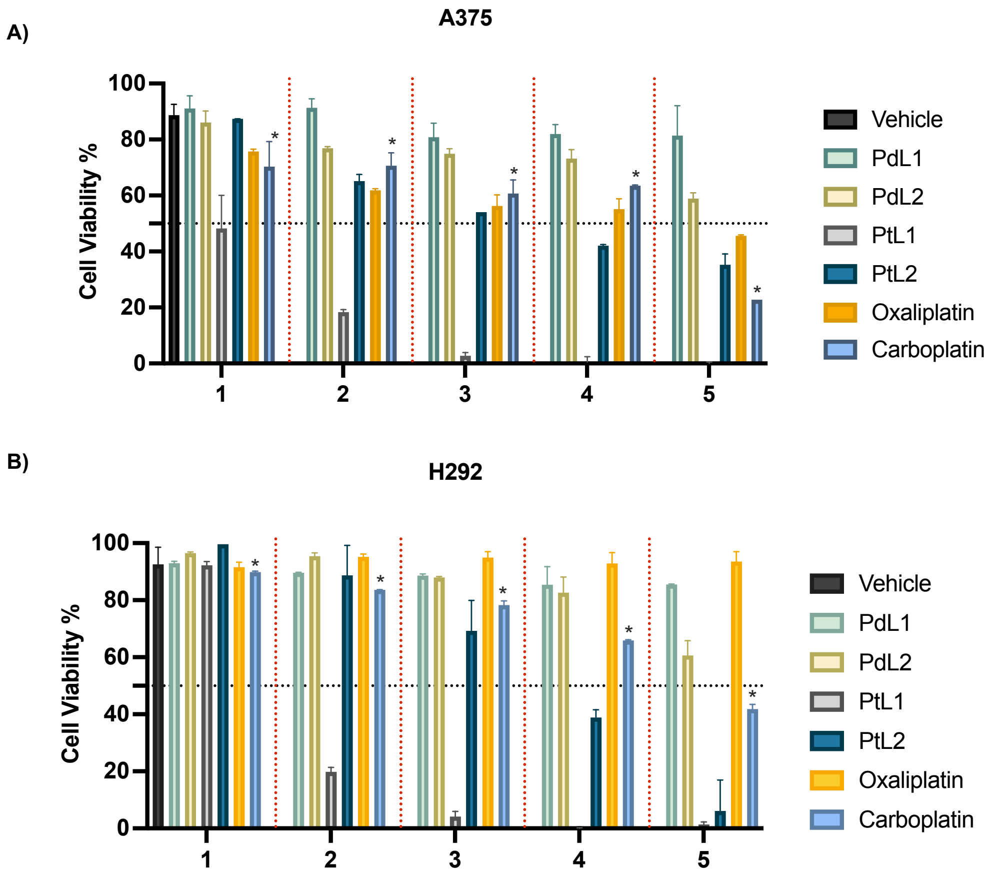

Figure S8 Dose-response evaluation of cytotoxic activity in A) A375 and B) H292 cells treated with metallosalen complexes and standard chemotherapeutics. Bar graphs show the percentage of cell viability in melanoma (A375, top) and non-small cell lung cancer (H292, bottom) cell lines after 20-hour treatments with Pd(II) and Pt(II) metallosalen complexes (PdL1, PdL2, PtL1, PtL2), oxaliplatin, and carboplatin at increasing concentrations (grouped as 1 to 5). Each group represents escalating compound doses, with metallosalens and oxaliplatin tested in the micromolar range (0.5 μM, 1.5 μM, 4.5 μM, 9.0 μM, and 13.5 μM), while carboplatin was evaluated in the millimolar range (0.5 mM to 13.5 mM) to reflect its potency. Vehicle controls (2% DMSO in media) were included for comparison. The dashed horizontal line marks the 50% viability threshold, and red dotted vertical lines separate concentration groups. *Note: Percent cell viability was calculated as: % Viability = 100 − % Cytotoxicity, relative to untreated controls. These values were used to construct dose–response curves for* IC_{50} *determination.*

	IC_{50}	
**Compounds**	**A375**	**H292**
PdL1	> 13.50 μM	> 13.50 μM
PdL2	> 13.50 μM	> 13.50 μM
PtL1	0.48 ± 0.07 μM	0.83 ± 0.08 μM
PtL2	4.00 ± 1.24 μM	4.19 ± 1.74 μM
Oxaliplatin	7.80 ± 4.83 μM	> 13.50 μM
Carboplatin	> 13.50 μM	10.65 ± 4.78 mM

Table S4. IC_{50} values (μM or mM) of metallosalens and standard chemotherapeutic agents oxaliplatin and carboplatin in A375 and H292 cell lines.

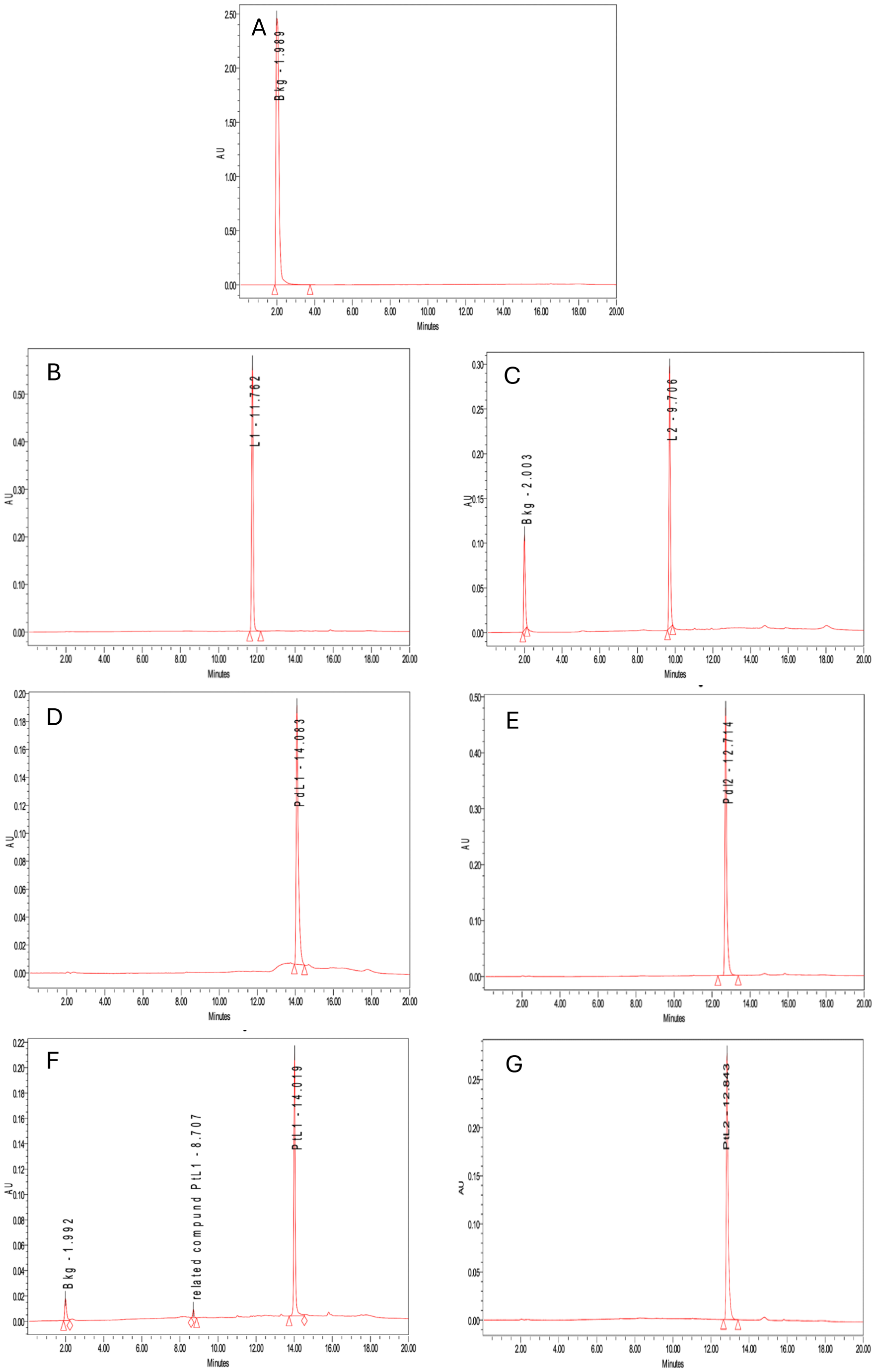

Figure S9. Analytical HPLC chromatograms of all ligands and metal complexes used in biological assays. Samples were prepared at a concentration of 25 ppm and injected in 50 µL volumes. Chromatographic separation was carried out on a C18 reversed-phase column (4.6 × 150 mm, 5 µm) under a linear gradient of acetonitrile and water, with a flow rate of 1.0 mL/min and a column temperature maintained at 25 °C. UV detection was performed at compound-specific wavelengths. All compounds showed purity ≥97%. Chromatograms include: (A) DMSO blank with a retention time of 1.989 min; (B) **L1** with a retention time of 11.762 min; (C) **L2** with a retention time of 9.706 min and DMSO at 2.003 min; (D) **PdL1** with a retention time of 14.083 min; (E) **PdL2** with a retention time of 12.714 min; (F) **PtL1** with a retention time of 14.019, DMSO at 1.992 min and a related compound at 8.707 min; and (G) **PtL2** with a retention time of 12.843 min.

Compounds	Retention Time (min)	Purity %
L1 (269 nm)	11.762	>99
L2 (247nm)	9.706	>99
PdL1 (286 nm)	14.083	>99
PdL2 (269 nm)	12.714	>99
PtL1 (246 nm)	14.019	97
PtL2 (277 nm)	12.843	>99

Table S5. HPLC Retention Times and Purity Percentages of L1, L2, PdL1, PdL2, PtL1, and PtL2. *Note: Retention times and calculated purity (%) were determined by analytical HPLC using a reversed-phase C18 column (4.6 × 150 mm, 5 μm), under a gradient of acetonitrile and water at a flow rate of 1.0 mL/min. Each compound was injected at 25 ppm (50 μL volume), and UV detection was performed at compound-specific wavelengths.*
